# The Impact of Chromate on *Pseudomonas aeruginosa* Molybdenum Homeostasis

**DOI:** 10.3389/fmicb.2022.903146

**Published:** 2022-05-24

**Authors:** Eve A. Maunders, Dalton H. Y. Ngu, Katherine Ganio, Sheikh I. Hossain, Bryan Y. J. Lim, Michael G. Leeming, Zhenyao Luo, Aimee Tan, Evelyne Deplazes, Boštjan Kobe, Christopher A. McDevitt

**Affiliations:** ^1^Department of Microbiology and Immunology, The Peter Doherty Institute for Infection and Immunity, University of Melbourne, Melbourne, VIC, Australia; ^2^School of Chemistry and Molecular Biosciences, The University of Queensland, Brisbane, QLD, Australia; ^3^Australian Infectious Diseases Research Centre, The University of Queensland, Brisbane, QLD, Australia; ^4^Institute for Molecular Bioscience, The University of Queensland, Brisbane, QLD, Australia; ^5^School of Life Sciences, University of Technology Sydney, Ultimo, NSW, Australia; ^6^Melbourne Mass Spectrometry and Proteomics Facility, Bio21 Molecular Science and Biotechnology Institute, The University of Melbourne, Melbourne, VIC, Australia

**Keywords:** *Pseudomonas aeruginosa*, solute-binding protein, molybdate, chromate, molybdenum, metal homeostasis, ABC transporter, ModA

## Abstract

Acquisition of the trace-element molybdenum *via* the high-affinity ATP-binding cassette permease ModABC is essential for *Pseudomonas aeruginosa* respiration in anaerobic and microaerophilic environments. This study determined the X-ray crystal structures of the molybdenum-recruiting solute-binding protein ModA from *P. aeruginosa* PAO1 in the metal-free state and bound to the group 6 metal oxyanions molybdate, tungstate, and chromate. *Pseudomonas aeruginosa* PAO1 ModA has a non-contiguous dual-hinged bilobal structure with a single metal-binding site positioned between the two domains. Metal binding results in a 22° relative rotation of the two lobes with the oxyanions coordinated by four residues, that contribute six hydrogen bonds, distinct from ModA orthologues that feature an additional oxyanion-binding residue. Analysis of 485 *Pseudomonas* ModA sequences revealed conservation of the metal-binding residues and β-sheet structural elements, highlighting their contribution to protein structure and function. Despite the capacity of ModA to bind chromate, deletion of *modA* did not affect *P. aeruginosa* PAO1 sensitivity to chromate toxicity nor impact cellular accumulation of chromate. Exposure to sub-inhibitory concentrations of chromate broadly perturbed *P. aeruginosa* metal homeostasis and, unexpectedly, was associated with an increase in ModA-mediated molybdenum uptake. Elemental analyses of the proteome from anaerobically grown *P. aeruginosa* revealed that, despite the increase in cellular molybdenum upon chromate exposure, distribution of the metal within the proteome was substantially perturbed. This suggested that molybdoprotein cofactor acquisition may be disrupted, consistent with the potent toxicity of chromate under anaerobic conditions. Collectively, these data reveal a complex relationship between chromate toxicity, molybdenum homeostasis and anaerobic respiration.

## Introduction

*Pseudomonas aeruginosa* is a Gram-negative opportunistic pathogen responsible for a wide range of human diseases, including pneumonia, septicemia, urinary tract and intestinal tract infections, and is associated with significant morbidity in immunocompromised individuals, such as those suffering from cystic fibrosis (*CF*; Nathwani et al., 2014). Although commonly associated with human infection, *P. aeruginosa* is also frequently isolated from environmental ecosystems, such as soils and water systems primarily associated with human activity (Crone et al., 2020). The environmental ubiquity of *P. aeruginosa* has been attributed to its relatively large genome, which encodes a diverse range of metabolic pathways and permits growth or survival in a range of chemically distinct, nutritionally limited niches. Importantly, *P. aeruginosa* is a facultative aerobe and, in the absence of oxygen, has the capacity to respire using nitrate or nitrite as alternative electron acceptors. The capacity for *P. aeruginosa* to persist and thrive in a diversity of chemical, oxygenic and non-oxygenic conditions has been linked to its persistence within complex microenvironments, such as the *CF* lung and polymicrobial biofilms (Schobert and Jahn, 2010; Arai, 2011; Cendra et al., 2019).

Anaerobic growth *via* dissimilatory nitrate reduction is coordinated through the molybdenum-cofactor-dependent enzyme complex NarGHI, which performs the initial step of reducing nitrate to nitrite (Bertero et al., 2003). Accordingly, acquisition of the transition element molybdenum (Mo) is essential for *P. aeruginosa* growth in anaerobic and microaerophilic environments *via* nitrate reduction (Pederick et al., 2014). Molybdenum, which predominantly occurs as the oxyanion molybdate (MoO_4_^2−^; henceforth MoO), is imported *via* the high-affinity ATP-binding cassette (ABC) permease, ModABC in *P. aeruginosa* (Rech et al., 1996; Pederick et al., 2014). In other bacterial species, molybdate uptake has also been reported to occur *via* a low-affinity molybdate-specific ABC permease MolABC (Tirado-Lee et al., 2011), sulfate/thiosulfate ABC permeases (Rosentel et al., 1995), and/or non-specific anion importers (Lee et al., 1990). The best characterized bacterial ModABC uptake system is from *Escherichia coli* (Rech et al., 1996; Hollenstein et al., 2007). Studies of *E. coli* ModABC established that MoO is acquired in the periplasm by the cluster D-IIIa solute-binding protein (SBP) ModA, which facilitates MoO transfer to the cytoplasmic membrane channel ModB. Transmembrane translocation of MoO then occurs in a process coupled to ATP hydrolysis *via* the nucleotide binding domain ModC (Rech et al., 1996; Hollenstein et al., 2007). Prior to utilization by Mo-dependent proteins, henceforth molybdoproteins, Mo is first incorporated into a molybdopterin molecule to produce the molybdenum cofactor (MoCo). The cofactor can be further modified for use by molybdoenzymes such as dimethyl sulfoxide reductase, xanthine oxidase, and sulfite oxidase (Iobbi-Nivol and Leimkühler, 2013; Leimkuhler, 2020). For NarGHI, the modified molybdenum cofactor is Mo-*bis* molybdopterin guanine dinucleotide (MGD; Bertero et al., 2003). Expression of the *modABC* operon and the MoCo biosynthetic genes are regulated by the Mo-dependent transcriptional regulator ModE, which negatively regulates expression in the presence of cytoplasmic Mo (McNicholas et al., 1996; Anderson et al., 2000).

Despite *E. coli* ModA having a high affinity for MoO (*K*_d_ = ~5–67 nM; Rech et al., 1996; Imperial et al., 1998; Ge et al., 2020), it has been shown to be permissive for binding to tungstate (WO_4_^2−^; henceforth WO), the oxyanion form of tungsten (W), a group 6 element with similar chemistry to molybdenum (Imperial et al., 1998). In *P. aeruginosa*, exposure to WO reduced MoO acquisition, which was attributed to competition between the two metal ions for the ModA SBP and the inappropriate sensing of W by ModE, thereby leading to repression of the *modABC* operon (Pederick et al., 2014). The result was a depletion in cellular Mo, with concomitant impacts on nitrate reductase activity and inhibition of anaerobic growth in WO-supplemented media (Pederick et al., 2014). Recently, *E. coli* ModA was shown to also interact with chromate (CrO_4_^2−^; henceforth CrO), the oxyanion form of the first group 6 element chromium (Cr), with a modest difference in relative affinity (*K*_d_ = ~68–162 nM) to MoO (Karpus et al., 2017). Industrial use of chromium is widespread due to its favorable properties in the production of metal alloys, pigments, textile dyes, and applications in tanneries. Anthropogenic factors have led to a substantial increase in the rate of CrO entry into the biosphere, with contamination of soils, sediments, groundwater, and aquatic systems presenting difficult health and bioremediation challenges (Cheung and Gu, 2007; Thatoi et al., 2014). Chromium can mediate significant toxicity within cells due to its flexible redox chemistry, which can lead to the generation of reactive oxygen species (ROS) and thereby damage DNA, proteins and cell membranes (Cervantes and Campos-Garcia, 2007; Cheung and Gu, 2007). Bacterial resistance to Cr toxicity has been attributed to DNA repair, detoxifying processes, and chromate efflux pumps, such as ChrA in Cr-resistant *P. aeruginosa* clinical strains.

In this study, we investigated the interaction of *P. aeruginosa* ModA with oxyanions of the group 6 elements: Cr, Mo, and W. By combining biochemical, structural, and molecular dynamic analyses of ModA-metal complexes, we show that the SBP is permissive for binding all three oxyanions at the primary metal-binding site. Despite the interaction of CrO with ModA, molecular microbiological and mass spectrometric analyses revealed that Cr intoxication occurred independently of Mo acquisition. Thus, CrO uptake in *P. aeruginosa* occurs by at least one alternative pathway. Sub-inhibitory CrO exposure dysregulated Mo homeostasis resulting in increased accumulation and altered distribution of the metal within the proteome. This manifested at a cellular level with CrO potently inhibiting *P. aeruginosa* growth on nitrate under anaerobic conditions. Collectively, these findings provide new insights into toxicity of CrO towards *P. aeruginosa* and reveal a complex relationship with Mo homeostasis and anaerobic respiration.

## Materials and Methods

### Cloning, Protein Expression, and Purification

High-fidelity PCR amplification of the mature coding sequence of *P. aeruginosa* PAO1 *modA* (residues 23–251, excluding the signal sequence) was carried out using primers listed in Supplementary Table S1. Ligation-independent cloning was used to insert the *modA* gene into an N-terminal hexahistidine (His6) tag-containing vector, pMCSG7, to generate pMCSG7-*modA*. The recombinant pMCSG7-*modA* vector was then used to transform *E. coli* BL21-Gold (DE3) competent cells. Strains and constructs are detailed in Supplementary Tables S2 and S3, respectively. High-level expression of the recombinant fusion ModA protein was achieved using auto-induction (Studier, 2005). An overnight cell culture (1 ml) was inoculated into 1 L of autoinduction media [10 g tryptone, 5 g yeast extract, 5 ml glycerol, 0.5 g glucose, 2 g α-lactose, 1 mM MgSO_4_, 25 mM (NH_4_)_2_SO_4_, 50 mM KH_2_PO_4_, 50 mM Na_2_HPO_4_, 100 μg.ml^−1^ ampicillin]. The cells were grown with vigorous shaking at 37°C to an optical density at 600 nm (OD_600_) of 0.4–0.8, before the temperature was reduced to 16°C for overnight growth and protein expression. The cells were then harvested *via* centrifugation at 5,180 × *g* for 30 min at 4°C. The cell pellets were resuspended in 100 ml binding buffer (50 mM HEPES pH 7.5, 500 mM NaCl, 30 mM imidazole) containing two Pierce protease inhibitor tablets (Thermo Fisher Scientific, United States), 6.6 mg of DNase I and 1 mM of sodium deoxycholate. The cells were lysed *via* sonication for two cycles using a Branson 450 Digital Sonifier (Branson Ultrasonics Corporation, United States) at 40% amplitude with 10 s pulses, 10 s rest, for a total sonication time of 1 min per cycle. The lysed cells were centrifuged at 22,716 × *g* for 45 min at 4°C. The supernatant was loaded onto a 5 ml HisTrap FF column (Cytiva, United States), which had been pre-equilibrated with binding buffer for immobilized metal affinity chromatography (IMAC). After washing with 50 ml binding buffer, the fusion His_6_-tagged ModA protein was eluted from the column with elution buffer (50 mM HEPES pH 7.5, 500 mM NaCl, 350 mM imidazole) and fractions were analyzed by denaturing SDS-PAGE. Fractions containing the fusion His_6_-tagged ModA protein were pooled together and placed in a SnakeSkin dialysis tubing 10 K MWCO (Thermo Fisher Scientific, United States) for overnight dialysis in dialysis buffer (10 mM HEPES pH 7.5, 150 mM NaCl, 0.5 mM EDTA, 1 mM DTT) at 4°C to remove the imidazole. A molar ratio of 20:1 tobacco etch virus (TEV) protease to His6-tagged ModA protein was also added to the tubing for cleavage of the fusion tag. After dialysis, the TEV-protease cleaved ModA protein solution was loaded onto 5 ml HisTrap™ FF column, which had been pre-equilibrated with binding buffer to remove the TEV protease and fusion tag. The protein solution was then loaded onto a HiLoad Superdex 75 pg 16/600 column (Cytiva, United States), which had been pre-equilibrated with gel filtration buffer (10 mM HEPES pH 7.5, 150 mM NaCl) and was connected to an ÄKTA Fast Protein Liquid Chromatography automated purification system (FPLC; Cytiva, United States) for size exclusion chromatography (SEC) at 4°C. Fractions were analyzed with denaturing SDS-PAGE and those that contained the purified ModA protein were pooled together, concentrated and stored at −80°C.

### Protein Crystallization, Crystal Structure Determination, and Structural Analyses

Crystals of metal-free ModA were obtained in 2 M (NH_4_)_2_SO_4_ and 0.1 M citrate (pH 3.5) at 20°C, with a protein concentration of 10 mg.ml^−1^, using the hanging-drop vapor-diffusion method. Due to difficulties in crystallizing the metal-bound ModA, chemical modification *via* methylation of surface lysine residues was performed on the metal-free ModA as previously described (Rayment et al., 1993; Rayment, 1997; Walter et al., 2006). Crystals of lysine-methylated metal-bound ModA were obtained at 293 K, using the hanging-drop vapor-diffusion method in the following conditions: chromate, 2.1 M (NH_4_)_2_SO_4_ and 0.1 M Tris–HCl pH 8.5, protein concentration of 6 mg.ml^−1^ and K_2_CrO_4_ at a 1:10 protein to CrO_4_^2−^ molar ratio; molybdate, 2.1 M (NH_4_)_2_SO_4_ and 0.1 M Tris–HCl pH 8.5, protein concentration of 8 mg.ml^−1^ and Na_2_MoO_4_ at a 1:10 protein to MoO_4_^2−^ molar ratio; tungstate, 2.2 M (NH_4_)_2_SO_4_ and 0.1 M Tris–HCl pH 8.5, protein concentration of 4 mg.ml^−1^ and Na_2_WO_4_ at a 1:10 protein to WO_4_^2−^ molar ratio. Prior to data collection, the harvested crystals were briefly soaked in solutions containing 20%–25% v/v glycerol as the cryoprotectant, before flash-cooling by rapid immersion in liquid nitrogen. The diffraction data were collected on single crystals at the Australian Synchrotron MX1 and MX2 beamlines (Aragão et al., 2018). To determine crystal structures, the diffraction data were indexed and integrated using *XDS* (Kabsch, 2010), then scaled and merged in *Aimless* (Evans and Murshudov, 2013). Initial phases were obtained by molecular replacement, using the tungstate-bound *Azotobacter vinelandii* ModA2 crystal structure as the search model in *Phenix.Phaser* (McCoy et al., 2007), followed by model building in *Phenix.AutoBuild* (Terwilliger et al., 2008). The structures were iteratively refined with *Phenix.Refine* (Afonine et al., 2012) and manually adjusted with *Coot* (Emsley et al., 2010). Structural analyses (superpositions and metal-ion coordination) were performed in MacPyMOL (v2.1 Schrödinger, LLC). Data collection, processing, and structure refinement statistics can be found in Supplementary Table S4.

### Bacterial Strains and Growth Media

The *P. aeruginosa* strains used in this study were the laboratory strain PAO1, and a PAO1 *modA* deletion strain generated by Pederick et al. (2014). Strains were grown in a semisynthetic cation-defined medium (CDM) containing 8.45 mM Na_2_HPO_4_, 4.41 mM KH_2_PO_4_, 1.71 mM NaCl, 3.74 mM NH_4_Cl, 0.5% (w/v) yeast extract (Bacto) and supplemented with vitamins (0.82 nM biotin, 1.62 μM nicotinic acid, 0.97 μM pyridoxine-HCl, 0.59 μM thiamine-HCl, 0.27 μM riboflavin and 0.1.26 μM calcium pantothenate), pH 6.8. Before use, media was Chelex-100 (Sigma-Aldrich) treated and supplemented with 0.1 mM CaCl_2_ and 2 mM MgSO_4_. Metal concentrations of CDM were determined by inductively coupled-plasma mass spectrometry (ICP-MS), with molybdenum, tungsten, and chromium present at ≤60 nM. Where stated, 100 μM (aerobic cultures) and 10 μM (anaerobic cultures) Na_2_MoO_4_ was added to CDM to generate molybdate-enriched CDM (M-CDM). Anaerobic cultures were supplemented with 100 mM KNO_3_, and where appropriate, media were supplemented with 320 μM K_2_CrO_4_ and Na_2_MoO_4_ for aerobic cultures and 32 μM K_2_CrO_4_ and Na_2_MoO_4_ for anaerobic cultures.

For growth analysis in aerobic conditions, overnight cultures of *P. aeruginosa* were standardized to an optical density at 600 nm (OD_600_) of 0.05 and grown for 24 h at 37°C, shaking at 240 rpm. For growth analysis in anaerobic conditions, cultures underwent an anaerobic shift as described in Pederick et al. (2014) and were standardized to OD_600_ = 0.05 in pre-reduced media. Anaerobic cultures were grown statically in a sealed anaerobic chamber with a GasPak EZ anaerobic sachet (Becton Dickinson, United States) for 24 h at 37°C.

### Whole-Cell Metal Content Determination

*Pseudomonas aeruginosa* was grown aerobically to an OD_600_ of 0.5 (mid-log phase), shaking at 240 rpm at 37°C. Anaerobic cultures were grown statically at 37°C for 13 h to approximately mid-log phase. The bacteria were washed twice with phosphate buffered saline (PBS) containing 5 mM ethylenediamineteraacetic acid (EDTA), and twice with PBS. Bacterial pellets were desiccated at 95°C overnight and digested in 250 μl 65% (v/v) HNO_3_ at 95°C for 20 min. Samples were centrifuged at 18,000 × *g* for 25 min and soluble material was diluted to a final concentration of 3.25% HNO_3_ using MilliQ-H_2_O. Elemental content was quantitatively analyzed in technical triplicate using an Agilent 8900 triple quadrupole ICP-MS (Agilent Technologies).

### Protein Metal Content Determination

Metal loading assays were performed on metal-free ModA (up to 30 μM) by mixing with 10-fold molar excess of Na_2_MoO_4_, Na_2_WO_4_, or K_2_CrO_4_. Metal loading assays were performed in 10 mM HEPES (pH 7.5) and 150 mM NaCl for 1 h at 4°C with agitation. An equimolar concentration of protein:EDTA was added during K_2_CrO_4_ loading to chelate Mg^2+^ ions. Unbound metal was removed by desalting on a PD10 column (GE Healthcare), and protein concentration was determined. Metal-free and metal-loaded proteins were prepared in 3.5% HNO_3_ and boiled for 15 min at 95°C. After cooling, samples were centrifuged for 20 min at 18,000 × *g*. The supernatant was analyzed by ICP-MS and the metal:protein ratio was determined.

### Ligand-Dependent Protein Mobility Shift Gel Electrophoresis

Ligand-dependent protein mobility shift gel electrophoresis under native PAGE conditions was performed as previously described (Arndt et al., 2012). Metal-free ModA (40 μM) was incubated with 10-fold molar excess of Na_2_HPO_4_, Na_2_SO_4_, Na_3_VO_4_, K_2_CrO_4_, Na_2_MoO_4_, and Na_2_WO_4_ (400 μM) for 2 h on ice. In microcentrifuge tubes, 10 μl of sample solution (0.187 M Tris.HCl pH 6.8, 30% glycerol, 80 μg.ml^−1^ Bromophenol Blue) was mixed with 10 μl of protein samples. The samples were loaded onto a continuous (15%) polyacrylamide gel and the gel tank was placed on ice in a cold room at 4°C. Gel electrophoresis was performed at 200 V for 2 h. After the run, the gel was stained with Coomassie Brilliant Blue for visualization of the relative mobilities of the ModA protein samples.

### Nano-Differential Scanning Fluorimetry

Metal-free ModA (100 μM) was incubated with 10-fold molar excess of K_2_CrO_4_, Na_2_MoO_4_, and Na_2_WO_4_ for 2 h on ice and centrifuged at 20,000 × *g* for 10 min at room temperature and transferred to new microcentrifuge tubes for the removal of any precipitated protein. They were then left to equilibrate at 23°C before loading into individual high sensitivity glass capillaries in triplicate. NanoDSF was performed using the Prometheus NT.48 (NanoTemper Technologies GmbH, Germany) at a temperature range of 20°C–95°C. Aliquots of purified metal-free ModA protein (100 μM) were preincubated with 10-fold excess oxyanions (1 mM of K_2_CrO_4_, Na_2_MoO_4_, and Na_2_WO_4_) for 2 h on ice. The samples were next centrifuged at 20,000 × *g* for 10 min at room temperature and transferred to new microcentrifuge tubes for the removal of any precipitated protein. They were then left to equilibrate at 23°C before loading into individual high sensitivity glass capillaries in triplicate. Thermal unfolding of ModA protein samples was determined based on fluorescence at 330 nm and 350 nm. Aggregation of samples was determined by backscattering.

### Isothermal Titration Calorimetry

Experiments were performed on a Nano ITC Low Volume isothermal titration calorimeter (TA Instruments, United States) at a temperature of 25°C. Forty micromolar metal-free ModA was loaded into the sample cell of the calorimeter and titrated with 320 μM of metal oxyanion ligand solutions (K_2_CrO_4_, Na_2_MoO_4_, and Na_2_WO_4_). An initial injection of 1 μl of ligand solution was followed by a series of 2 μl titrations with an injection interval of 200 s for a total of 25 cycles. Data were analyzed using the NanoAnalyze Data Analysis software (v3.10.0, TA Instruments, United States). Enthalpy changes were corrected by subtracting the background mean enthalpies from the raw titration data and data were then normalized with the ligand concentrations. Excluding the first data point, an “independent” model for a single binding site for the ligands was used to fit the data.

### Comparative Genomics

A database of 522 publicly available *Pseudomonas* genomes was screened for molybdenum-associated genes by the BLASTN screening tool, Screen Assembly (v1.2.7; Davies et al., 2019), applying cut-offs of 80% identity and 80% reference length. Gene absences were validated by screening 3× 200 bp segments of target genes, to account for genes with overall low sequence similarity or disrupted by contig breaks (partial matches). Nucleotide and translated protein sequences were used for variation analysis by MUSCLE alignment (v3.8.425; Edgar, 2004). Aligned nucleotide sequences were trimmed using Noisy (v1.5.12.1; Dress et al., 2008) and a phylogenetic tree was constructed using the maximum likelihood approach with IQ-Tree incorporating 1,000 ultrafast bootstrap resamplings (Galaxy v2.1.2; Minh et al., 2020). Trees were visualized using iTOL (v6.1; Letunic and Bork, 2021).

### Molecular Dynamics Simulations

Chain A in the structure of the open, metal-free conformation of ModA was extracted from the asymmetric unit of the crystal structure and all crystallographic waters, glycerol and sulfate ions were removed. All His residues were described in their protonated form based on hydrogen bonding potential with surrounding residues. The protein structure was placed in a rectangular box with at least 1.2 nm between the protein and the box wall, followed by solvation with water molecules. Sodium and chloride ions were added to neutralize the charge on the protein and to reach a final ionic strength of 100 mM NaCl. The final system, which consisted of 60,911 atoms, was energy minimized using the steepest descent algorithm. The system was relaxed using a 5-ns NVT followed by a 5-ns NPT run. Three independent simulations of 750 ns each were performed.

Simulations were carried out using GROMACS 2020 (Abraham et al., 2015; Lindahl et al., 2020). The protein and ions were described with parameters in the GROMOS 54a7 force field (Schmid et al., 2011) and for water the simple point charge (SPC) model was used (Berendsen et al., 1981). Periodic boundary conditions were applied in all dimensions. Temperature was maintained at 298 K using a V-rescale thermostat with a time constant of 0.1 ps, and separate coupling for the protein and the solvent (water/ions). Pressure was kept at 1 bar with a Parrinello-Rahman barostat (Parrinello and Rahman, 1981) using isotropic coupling and a time constant of 2.0 ps and an isothermal compressibility of 4.5 × 10^−5^ bar^−1^. A Verlet scheme with cut-offs 1.0 nm and 1.2 nm was used for van der Waals and short-range interactions (Páll and Hess, 2013). Long-range electrostatics were treated with a Particle-mesh Ewald algorithm with a grid spacing 0.16 and a cubic interpolation of 4 (Darden et al., 1993). The LINCS constraints algorithm was used for constraints (Hess et al., 1997). Initial velocities were assigned from Maxwellian distributions at 298 K using different random seeds for the three independent runs.

Analysis was carried out using GROMACS tools and the python library MDAnalysis (Michaud-Agrawal et al., 2011). Equilibration of the system was assessed using RMSD vs. time calculated using all backbone atoms in the protein. Unless otherwise stated, analysis was carried out using the last 250 ns from the trajectory of each independent simulation. Images were prepared using VMD (Humphrey et al., 1996). Cluster analysis was performed using the algorithm of Daura et al. (1999) as implemented in the GROMACS tools gmx cluster. A backbone neighbor RMSD cut-off of 2.5 Å was used.

### Liquid Chromatography-Inductively Coupled Plasma-Mass Spectrometry

*Pseudomonas aeruginosa* cell pellets were washed once with PBS before resuspension in 100 μl 20 mM Tris–HCl, pH 8.0 and lysed using a Bioruptor (25 cycles; 30 s on, 30 s off) at 4°C. Cell lysates were clarified by centrifugation at 16,000 × *g* for 15 min at 4°C to remove insoluble material. The supernatant was collected, and 100 mg of total protein fractionated by anion exchange (AEX) chromatography using a Bio SAX NP3 HPLC column (4.6 × 50 mm column; Agilent Technologies) on a Bio-Inert Infinity 1260 HPLC (Agilent Technologies). The AEX column was equilibrated in Buffer A (20 mM Tris.HCl pH 8.0) and proteins eluted using a linear gradient of 0%–100% Buffer B (20 mM Tris.HCl pH 8.0, 1 M NaCl) at a flow rate of 0.4 ml.min^−1^. Fractions (0.4 ml) were collected at intervals of 1 min. AEX fractions were then individually separated *via* SEC in 100 mM ammonium nitrate, pH 7.5 at a flow rate of 0.4 ml.min^−1^ on a Bio SEC-3 HPLC column (4.6 × 300 mm, 3 μm particle size, 150 Å pore size; Agilent Technologies) *via* a Bio-Inert Infinity 1260 HPLC hyphenated to an 8,900 triple quadrupole ICP-MS (Agilent Technologies). Elemental distribution maps were generated by aggregating the SEC retention times and individual metal intensities from individual AEX fractions into a data frame that contained fraction number, retention time and detected abundance for each point. Visualizations were produced with Python (v3.9.9) using Matplotlib (v3.5.0).

### Data Availability

The accession codes for the structures deposited in the Protein Data Bank are as follows: 7T4Z (metal-free ModA), 7T50 (chromate-bound ModA), 7T51 (molybdate-bound ModA), and 7T5A (tungstate-bound ModA).

## Results

### ModA Can Bind Chromate

**Figure 1:**
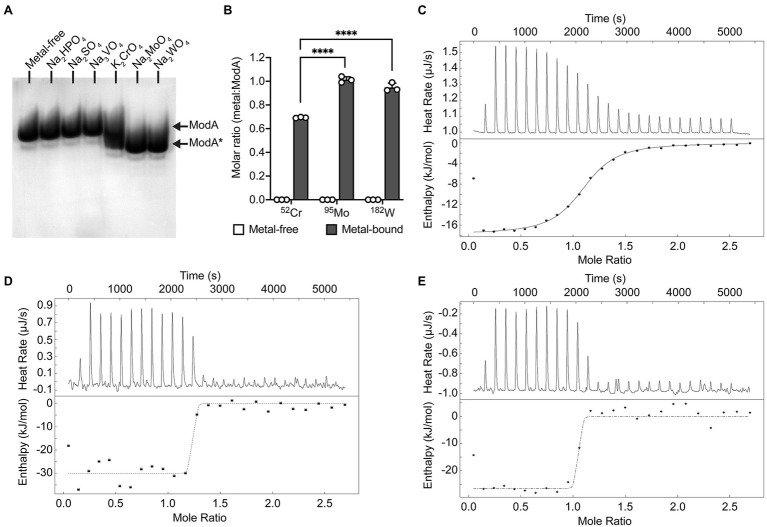
Biochemical characterization of *Pseudomonas aeruginosa* ModA. **(A)** Ligand-dependent protein mobility shift gel electrophoresis was performed under native PAGE conditions. Recombinant *P. aeruginosa* PAO1 ModA was incubated with 10-fold molar excess of Na_2_HPO_4_, Na_2_SO_4_, Na_3_VO_4_, K_2_CrO_4_, Na_2_MoO_4_, and Na_2_WO_4_. Ligand-free (ModA) and ligand-bound (ModA*) states are indicated by arrows. **(B)** ICP-MS analysis of recombinant ModA incubated with 10-fold molar excess of Na_2_MoO_4_, Na_2_WO_4_, and K_2_CrO_4_. Metal content is expressed as molar ratio of metal to ModA. Data represent the mean (±SEM) of three independent biological experiments. Statistical significance of the differences was determined by two-way ANOVA with Sidak post-test, **** = *p* < 0.0001. **(C–E)** Isothermal titration calorimetry (ITC) curves of 40 μM ligand-free ModA titrated against 320 μM of chromate **(C)**, molybdate **(D)**, or tungstate **(E)** oxyanion ligands. Raw titration data (top panel) and complete binding isotherms for ModA (bottom panel) are shown. The integrated curves, after correction for enthalpy changes and normalizing the oxyanion ligand concentrations, were best fit using the “independent” model and a single binding site for the ligands (line).

Recombinant *P. aeruginosa* ModA was previously shown to have the capacity to interact with CrO (Fernández et al., 2019). To further investigate this observation, 100 μM purified, recombinant, metal-free ModA was incubated with 10-fold excess (i.e., 1 mM) of K_2_CrO_4_; the known ligands, Na_2_MoO_4_ and Na_2_WO_4_; and other known non-interacting oxyanions, Na_2_HPO_4_, Na_2_SO_4_, and Na_3_VO_4_. Ligand-dependent protein mobility shift gel electrophoresis, which reports on overall changes in the size, mass, and charge of the protein, was performed under non-denaturing conditions. This revealed the metal oxyanion ligands CrO, MoO, and WO substantially altered the migration pattern of ModA, in contrast to phosphate, sulfate, and vanadate, which only exerted minor influences on the protein. Taken together, these data suggest the formation of an oxyanion ligand-ModA protein complex with altered migration properties (Figure 1A). The ModA-ligand complexes were then analyzed by ICP-MS (Figure 1B). ModA bound CrO with 0.69 ± 0.01 mol ^52^Cr.mol ModA^−1^, although this was significantly less than MoO, 1.01 ± 0.03 mol ^95^Mo.mol ModA^−1^, or WO, 0.95 ± 0.04 mol ^182^W.mol ModA^−1^. To complement these analyses, nano-differential scanning fluorimetry (nanoDSF) was performed. NanoDSF provides indicators of protein thermostability that include the onset temperature (*T*_onset_) and midpoint transition temperature (*T*_m_) for global conformational unfolding, and the temperature at which aggregates are first detected (*T*_agg_). Analysis of metal-free and metal-bound ModA revealed that the CrO-bound complex had +12.6°C change in the midpoint transition temperature (Δ*T*_m_), relative to the metal-free protein (Table 1). However, this was substantially less than MoO- and WO-bound ModA complexes (Table 1). A similar trend was also observed for the *T*_onset_ and *T*_agg_ values, with CrO inducing a positive shift in values relative to metal-free ModA, but less than the MoO- and WO-bound ModA complexes (Table 1). Isothermal titration calorimetry (ITC) experiments with the metal oxyanion ligands were also performed (Figures 1C–E; Table 2). This revealed that ModA bound MoO and WO with comparable affinity, with equilibrium dissociation constant (*K*_d_) values of 1.0 ± 1.3 and 1.2 ± 2.0 nM, respectively (Figures 1D,E; Table 2). Notably, these *K*_d_ values for MoO are higher than those reported for orthologous ModA proteins from *E. coli* (*K*_d_ = ~5–67 nM; Rech et al., 1996; Imperial et al., 1998; Ge et al., 2020), *Pseudomonas fluorescens* N2E2 (*K*_d_ = 27 ± 6.2 nM; Ge et al., 2020), and *Bacillus* strain EB106-08-02-XG196 (*K*_d_ = 2.2 ± 1.0 nM; Ge et al., 2020). For CrO, *P. aeruginosa* ModA had a *K*_d_ value of 927.0 ± 87.7 nM, approximately 900-fold lower than MoO and WO (Figure 1C; Table 2). Thus, the relative affinity of *P. aeruginosa* PAO1 ModA for CrO is more similar to *Bacillus* strain EB106-08-02-XG196 ModA (*K*_d_ = 1.56 ± 0.5 μM; ~750-fold difference in affinity for CrO vs. MoO; Ge et al., 2020), than *E. coli* (*K*_d_ = 68–162 nM; ~2–4 fold difference in affinity for CrO vs. MoO; Karpus et al., 2017). Collectively, these data show that *P. aeruginosa* ModA has the capacity to interact with the oxyanions of group 6 metals and, notably, with greater affinity for MoO and WO than reported for orthologous ModA SBPs to date. To further investigate the interaction of *P. aeruginosa* ModA with oxyanion ligands, crystal structures of the SBP in the metal-free and metal-bound states were determined.

**Table 1:** NanoDSF analyses of metal-free and metal-bound ModA.

Calculated parameters	Metal-free ModA	CrO-ModA	MoO-ModA	WO-ModA
*T*_onset_ (°C)	49.0	58.4	78.9	79.2
*T*_m_ (°C)	51.4	64.0	85.6	86.0
*T*_agg_ (°C)	47.2	57.8	74.3	74.7
Δ*T*_m_ (°C)	–	+12.6	+34.2	+34.6
Δ*T_agg_* (°C)	–	+10.6	+27.1	+27.5

**Table 2:** ITC analyses of metal-free and metal-bound ModA.

Calculated parameters	Metal-free ModA	CrO-ModA	MoO-ModA	WO-ModA
ΔH (kJ.mol^−1^)	–	−17.7 ± 0.2	−30.1 ± 2.0	−26.6 ± 1.5
ΔS (J.(mol.K)^−1^)	–	56.1	71.5	81.5
*K*_d_ (nM)	–	927.0 ± 87.7	1.0 ± 1.3	1.2 ± 2.0
*n*	–	1.1 ± 0.0	1.2 ± 0.0	1.0 ± 0.0

### Structural Analyses of Metal-Free ModA

**Figure 2:**
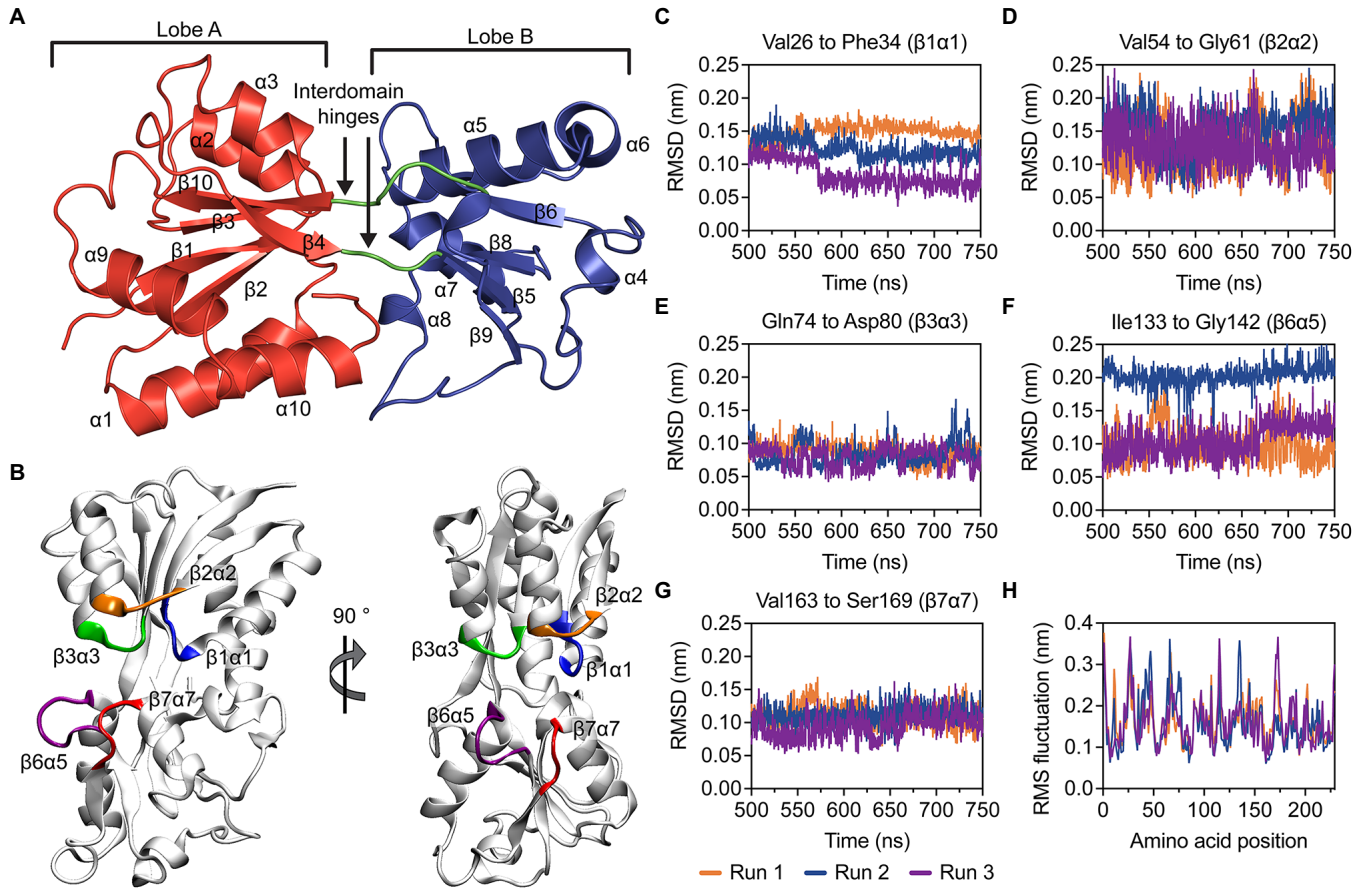
Structural and molecular dynamics simulations analyses of metal-free *P. aeruginosa* ModA. **(A)** Cartoon representation of metal-free *P. aeruginosa* PAO1 ModA. Lobe A, lobe B and the interdomain hinges are shown in red, blue, and green, respectively. The secondary structures of the protein are assigned and labelled. **(B)** Dominant conformations of the metal-free ModA protein, determined by clustering analysis. Flexibility of the loops β1α1 (blue), β2α2 (orange), β3α3 (green), β6α5 (purple), and β7α7 (red) was analyzed by measuring rmsd over time **(C–G)**. **(H)** RMS fluctuations of the residues in ModA. Data was calculated in triplicate 750 ns simulations and the data for each simulation is shown.

The crystal structure of recombinant, mature *P. aeruginosa* PAO1 ModA (residues 23–251) in the open, metal-free conformation was determined at 1.78 Å resolution (Figure 2A; Supplementary Table S4). There are two molecules in the asymmetric unit, chains A and B, with chain A used for structural analyses and comparisons. ModA has a fold characteristic of cluster D-IIIa SBPs and is made up of a bilobal structure separated by a deep cleft. As the polypeptide chain passes back and forth between the two domains, this results in both the N- and C-termini located within lobe A and creates two flexible hinges connecting the two lobes, comprised by residues Gly104-Thr105 and His209-Ile212. Each lobe contains a central five-stranded mixed β-sheet surrounded by five α-helices. Using the nomenclature described previously (Richardson, 1981), the β-sheet connectivity for lobe A is −1x, +2x, +2x, −1, and +2, +1x, −2x, −2x for lobe B.

Molecular dynamics (MD) simulations were used to investigate the stability of the open, metal-free ModA structure. The open, metal-free state of ModA was simulated for 750 ns in triplicate and the last 250 ns of each trajectory were used for analysis. Overall, the metal-free structure was stable and did not exhibit any large conformational changes. This is reflected in cluster analysis, where all structures from a given trajectory were found within the same cluster using a backbone neighbor root mean square deviation (rmsd) cut-off of 2.5 Å. The conformational freedom of the βα loops surrounding the binding site (β1α1, β2α2, β3α3, β6α5, and β7α7; Figure 2B) were analyzed by measuring the rmsd values over time (Figures 2C–G). These analyses showed that there was limited movement in the residues of the metal-binding pocket, which may be attributed to the relatively short length of the βα loops. Of the five binding site loops, β3α3 exhibited a very low level of mobility. Interestingly, this was the only loop that was not found to contain a putative metal binding residue. Further, analysis of the root-mean square fluctuations (rmsf; Figure 2H) showed that none of the loops or other structural elements showed a particularly high level of mobility. Overall, the MD simulations suggest that the open, metal-free state of ModA is not comprised by regions of significant flexibility and its conformational ensemble is centered around a stable protein fold that is very similar to the crystal structure.

### Structural Analyses of Metal-Bound ModA

**Figure 3:**
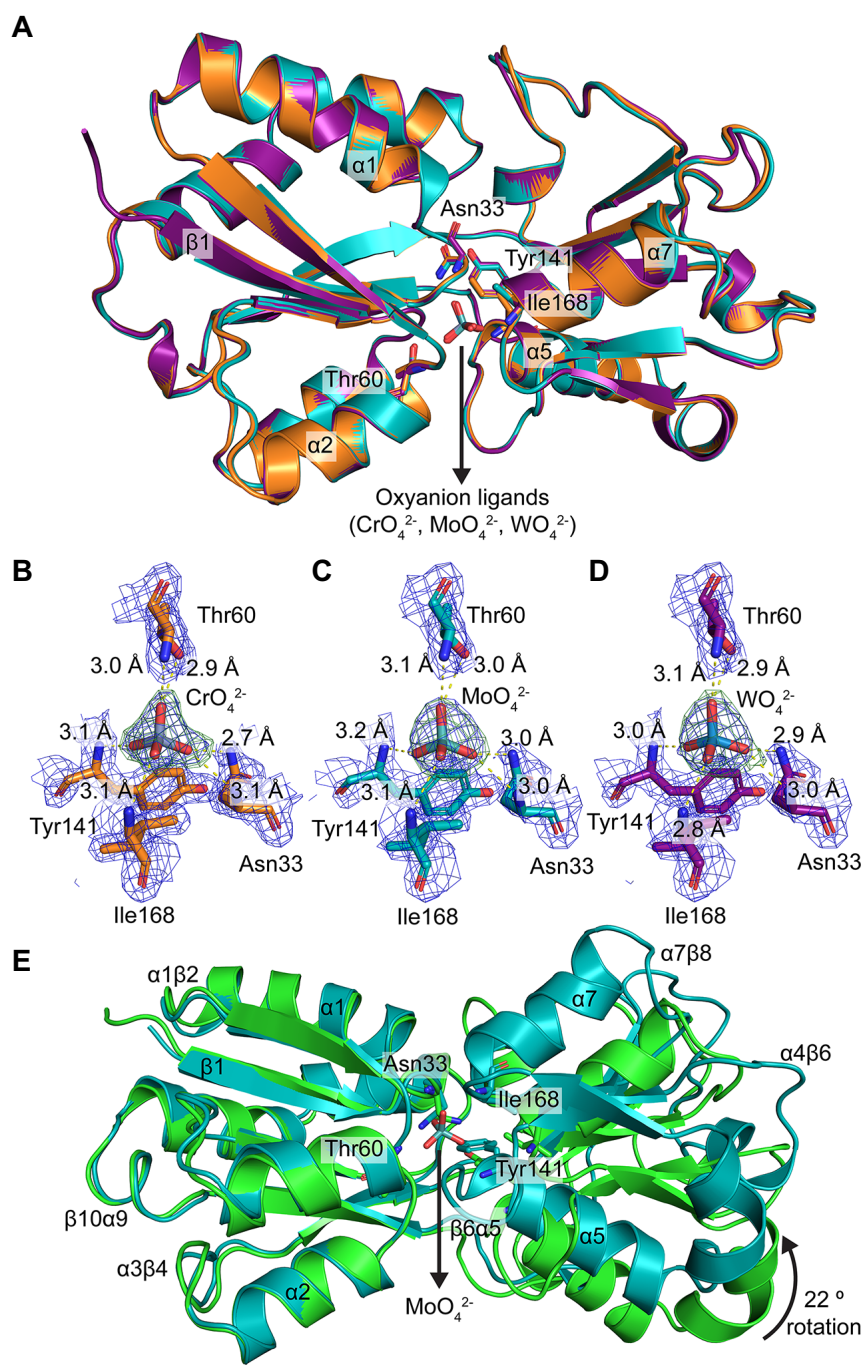
Structural characterization of metal-bound *P. aeruginosa* ModA. **(A)** Structural superposition of the crystal structures of metal [chromate (orange), molybdate (teal), tungstate (purple)]-bound ModA in cartoon representation. Some of the secondary structures are assigned and labelled. The coordinating residues and metal oxyanion ligands are shown in stick representation and labelled. The nitrogen and oxygen atoms are colored blue and red, respectively. **(B–D)** Hexacoordinate hydrogen bonding of **(B)** chromate, **(C)** molybdate, and **(D)** tungstate at the binding site. The hydrogen bonds are shown as yellow dotted lines and labelled with their respective bond lengths. The metals and residues are shown in stick representation and labelled. The 2mF_o_-DF_c_ electron density maps of the coordinating residues and metals are colored in dark blue and contoured at 1.5 σ, within a 2.0 Å radius around each residue. The polder (OMIT) electron density maps of the metals are colored in dark green and contoured at 4.0 σ, within a 2.0 Å radius around the ligand. **(E)** Structural superposition of the crystal structures of metal-free (green) and molybdate-bound (teal) ModA in cartoon representation. Some of the secondary structures and loops are assigned and labelled. The coordinating residues and metal are shown in stick representation and labelled. The nitrogen and oxygen atoms are colored blue and red, respectively.

The crystal structures of ModA in closed, metal-bound conformations were determined at 1.90, 2.50, and 2.16 Å, for CrO-, MoO-, and WO-bound ModA, respectively (Figure 3A; Supplementary Table S4). Similar to the metal-free ModA crystal structure, two molecules were present in the asymmetric unit of each complex structure and chain A was selected for structural analyses and comparisons. Superposition of the Cα traces of the metal-bound crystal structures showed that the overall geometry of all three structures is maintained, with rmsd values of 0.14–0.20 Å between any two structures analyzed. In all three structures, the metal-binding site is positioned deep at the interface between the two lobes and forms a buried pocket that accommodates a single CrO, MoO, or WO oxyanion. Two residues from each lobe are involved in hexacoordinate hydrogen bonding to the metal oxyanion: Asn33 (β1α1 loop) and Thr60 (β2 strand) from lobe A; and Tyr141 (α5 helix) and Ile168 (α7 helix) from lobe B. All four residues are involved in main-chain amide (NH) interactions, while Asn33 and Thr60 are also involved in side-chain amide and hydroxy (OH) interactions, respectively. The electrostatic hydrogen bonds between the residues and metal oxyanions are very similar in length, ranging from 2.8 to 3.2 Å (Figures 3B–D). The metal-binding pocket also reveals that there are seven residues involved in hydrophobic interactions with the metal oxyanion: Ala31 (β1 strand), Ala32 (β1α1 loop), Ala59 (β2α2 loop), Ala79 (β3α3 loop), Ala139 (β6α5 loop), Pro140 (α5 helix), and Asn167 (β7α7 loop).

The combination of structures also permits direct comparison of metal-free *P. aeruginosa* ModA with oxyanion-bound complexes. However, due to the lack of force-fields available for the metal oxyanions, MD simulations could not be performed. Structural superposition of the Cα traces of open, metal-free ModA with the closed, metal-bound states reveals rmsd values of 2.57 Å (227 residues), 2.52 Å (227 residues), and 2.57 Å (228 residues) for MoO-, CrO-, and WO-bound ModA, respectively. With lobe A fixed in place, metal-binding induces lobe B to undergo a relative rotation of ~22°, completely occluding the metal-binding cleft to bulk solvent. This rotation of lobe B also repositions outer loops (α1β2, α3β4, α4β6, α7β8, and β10α9) and the metal-binding residues Asn33 and Tyr141 (Figure 3E). Metal-binding also results in the α1 and α2 helixes from lobe A moving approximately 3° closer together, while the α5 and α7 helixes from lobe B moving approximately 5° further apart. Overall, the conformational changes associated with the ModA metal-binding mechanism are similar to those reported for the *E. coli L*-arabinose-binding protein (Mao et al., 1982). Thus, *P. aeruginosa* ModA appears to employ a “Venus’ fly-trap” mechanism for oxyanion binding.

In the metal-free conformation, the two lobes are stabilized in the open conformation by four hydrogen bonds (Asn33-Ser188, Tyr141-Gln214, Ser188-Tyr249, and Gly194-Ser246; Supplementary Figure S1A). Upon MoO-binding, two of the hydrogen bonds (Asn33-Ser188 and Gly194-Ser246) are broken and two additional hydrogen bonds are formed to stabilize the closed conformation: one between the metal-coordinating residues Asn33 and Tyr141, and one between Gly61 and Thr138 (Supplementary Figure S1B). The latter closes the binding site entrance, occluding access to bulk solvent. Notably, the two new hydrogen bonds formed (Asn33-Tyr141 and gating Gly61-Thr138) are maintained in all three metal-bound structures, and the other two hydrogen bonds (Tyr141-Gln214 and Ser188-Tyr249) are also present in the metal-free structure. Together, these analyses highlight how minor structural differences between the MoO-, WO-, and CrO-bound conformations impact the intra-protein interactions and potentially influence protein stability, flexibility, and function. Collectively, these data show that the chemistry of the *P. aeruginosa* ModA binding site is permissive for interaction with CrO, MoO, and WO. This promiscuous interaction with group 6 oxyanions is facilitated by binding site residues Asn33, Thr60, Tyr141, and Ile168, coupled with conformational and bonding rearrangements that tightly secure the bound metal within the SBP.

### Bacterial ModA Proteins Are Structurally Conserved Despite Limited Sequence Similarity

A multiple sequence alignment (MSA) of *P. aeruginosa* PAO1 ModA with six orthologous bacterial ModA proteins with available structural data was constructed for sequence and structural comparisons (Supplementary Figure S2). Structural superposition of the Cα traces of metal-free *P. aeruginosa* ModA with metal-free ModA orthologues from *Yersinia pestis* [PDB ID: 6NIO, rmsd value of 1.98 Å (198 residues)] and *Clostridioides difficile* [PDB ID: 4KD5, rmsd value of 1.92 Å (198 residues)] show that in the metal-free state, the SBPs adopt conformations that expose the metal-binding site to bulk solvent (Supplementary Figure S3A). Structural superposition of the Cα traces of the MoO-bound *P. aeruginosa* ModA with metal-bound ModA orthologues from *A. vinelandii* (PDB ID: 1ATG, rmsd value of 0.78 Å; 208 residues; Lawson et al., 1998), *Xanthomonas axonopodis* pv. *citri* [PDB ID: 2H5Y, rmsd value of 1.03 Å (184 residues)] *Vibrio cholerae* [PDB ID: 4RXL, rmsd value of 0.86 Å (170 residues)] and *E. coli* (PDB ID: 1AMF, rmsd value of 0.89 Å; 170 residues; Hu et al., 1997; Lawson et al., 1997, 1998; Balan et al., 2008) shows that they all adopt a closed, metal-bound conformation, with the metal oxyanions located within a bulk solvent occluded binding site (Supplementary Figure S3B). Interestingly, analysis of the binding site reveals that these orthologous ModA structures have a fifth residue involved in hydrogen binding to the metal *via* a side-chain OH interaction that is not present in *P. aeruginosa* PAO1 ModA (Hu et al., 1997; Lawson et al., 1997; Balan et al., 2008; Supplementary Figure S4). However, these analyses also show substantial variation in composition of the ModA binding site, indicating that the metal-coordinating residues are not strictly conserved across species, and this may contribute to the breadth of affinity values reported for interaction with MoO. Despite this, the low rmsd values (<2.0 Å) suggest that the cluster D-IIIa SBP fold is structurally conserved amongst bacterial ModA proteins, with the distinct SBPs adopting similar conformations in the open, metal-free and closed, metal-bound states.

### ModA Is Highly Conserved Amongst *Pseudomonas aeruginosa* Strains

*Pseudomonas* is one of the most highly diverse bacterial genera known, with environmental representatives known to possess high levels of diversity in terms of molybdoenzyme number and type (Leimkuhler and Iobbi-Nivol, 2016). It logically follows that MoO acquisition is crucial for these species and so the prevalence and conservation of *P. aeruginosa* PAO1 ModA in *Pseudomonas* species was investigated. Here, a database of 522 publicly available *Pseudomonas* genomes, comprising 222 human isolates, of which 199 were *P. aeruginosa*, and 300 environmental isolates (Supplementary Table S5), was constructed. Using the PAO1 *modA* gene as a reference sequence, putative *modA* genes were identified in all *P. aeruginosa* human isolates analyzed, with a high sequence conservation of 99.2% pairwise identity. PAO1 *modA* shared 98.9% pairwise identity with the human isolate *P. aeruginosa modA* consensus sequence, indicating that PAO1 is a suitable representative strain for the characterization of *P. aeruginosa* ModA. Initially, only 48 non-*aeruginosa* strains were found to possess a *modA* sequence that satisfied the screening threshold (80% identity and 80% sequence length). With this, the non-*aeruginosa* and environmental *Pseudomonas* isolate genomes were re-screened using *modA* reference sequences from 16 different major clades of environmental *Pseudomonas* species. This identified a further 238 genomes containing full-length putative *modA* sequences (Supplementary Table S5), and 59 genomes that contained partial matches to the *modA* sequences, bringing the total carriage of *modA* in the 522 *Pseudomonas* genomes to 95.0%.

**Figure 4:**
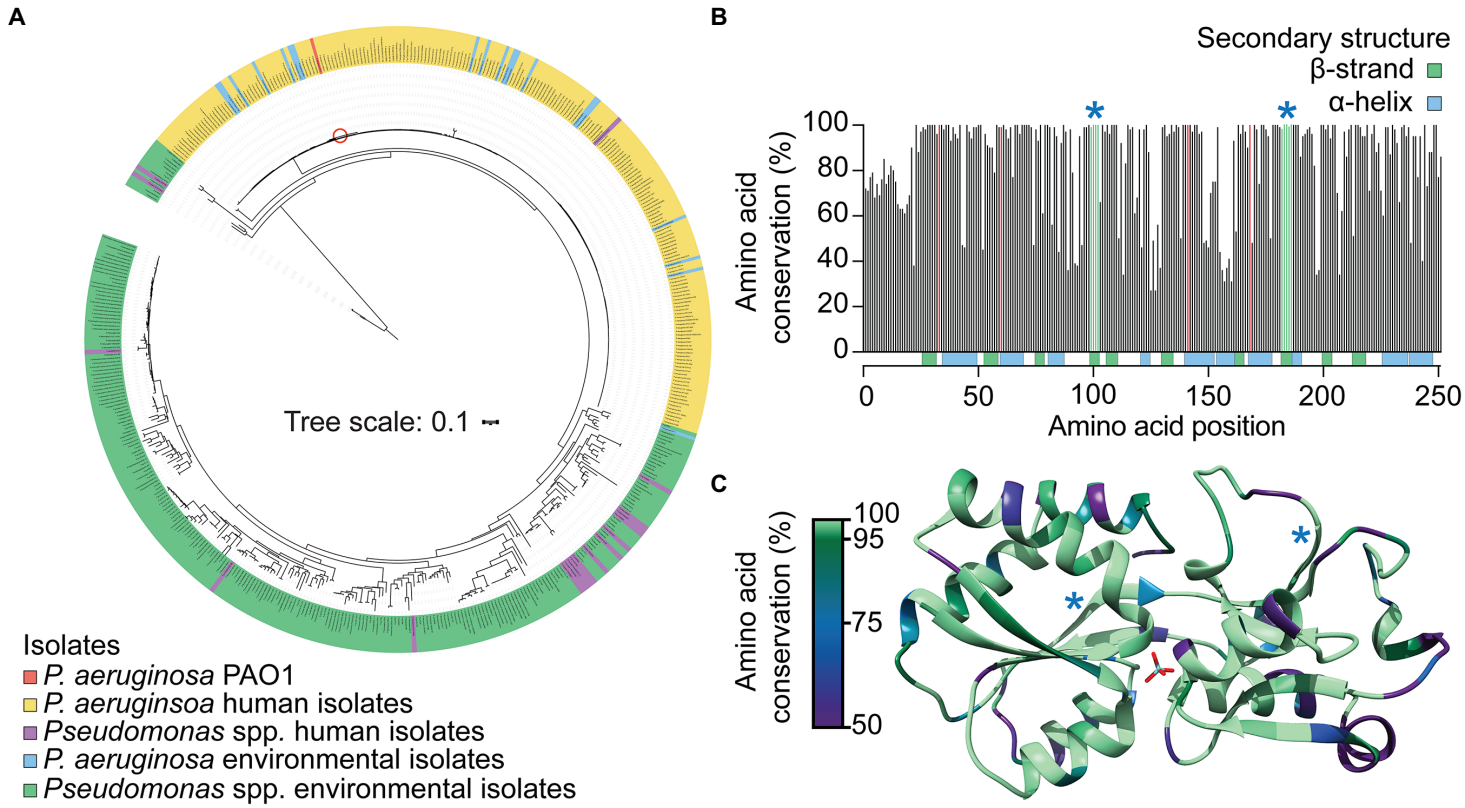
Conservation of ModA in *Pseudomonas* species. **(A)** Phylogenetic analysis of *modA* nucleotide sequences from 522 publicly available *Pseudomonas* spp. genomes. Environmental and human isolates are as indicated and PAO1 indicated by a red circle. The translated ModA sequences were used to: **(B)** determine the frequency of amino acid conservation at each position within the protein; and **(C)** map conservation to the tertiary structure of *P. aeruginosa* MoO-bound ModA. Metal-coordinating residues are indicated by red bars **(B)** and conserved β-strands are indicated by green bars and blue asterisks **(B)** or blue asterisks **(C)**.

Phylogenetic analysis of the 485 full-length *modA* nucleotide sequences highlighted the substantial variation observed in environmental isolate strains sequences (61.5% pairwise identity), and the conservation among *P. aeruginosa* isolates, irrespective of isolation source (Figure 4A). Only the *P. fluorescens* strain NCTC10783 clustered closely with *P. aeruginosa* isolates. However, NCBI taxonomic analysis determined *P. aeruginosa* as the best matching type-strain for this genome, which may suggest possible isolate misclassification. Despite high nucleotide sequence variability, the translated ModA sequences shared 84.0% pairwise identity, with 138 of the 251 amino acid residues of *P. aeruginosa* PAO1 ModA conserved in ≥95% of the *Pseudomonas* species analyzed. Regions of high conservation corresponded with residues of the metal-binding site (Figure 4B; red bars), within which the putative binding residues Asn33, Tyr141 and Ile168 were conserved in 100% of ModA sequences and Thr60 in 99.2%. This suggests that despite the diversity of binding site chemistry observed in other bacterial ModA proteins, within the *Pseudomonas* population the metal-binding site is highly conserved. Regions of high conservation were also observed in β-strand-forming residues, with β4 and β8 conserved in ≥98% of sequences (Figures 4B,C; green bars and blue asterisks). This suggests that these β-sheets play a crucial structural role, possibly serving to stabilize to the adjoining metal-binding site. In summary, these analyses reveal that ModA is widely carried in *Pseudomonas* species and, although there is substantial diversity within the gene, the overall tertiary structure and metal-binding site are highly conserved. It therefore follows that ModA-mediated MoO uptake is an important facet for the survival and propagation of both pathogenic and environmental pseudomonads.

### Anaerobiosis Increases Chromate Susceptibility

**Figure 5:**
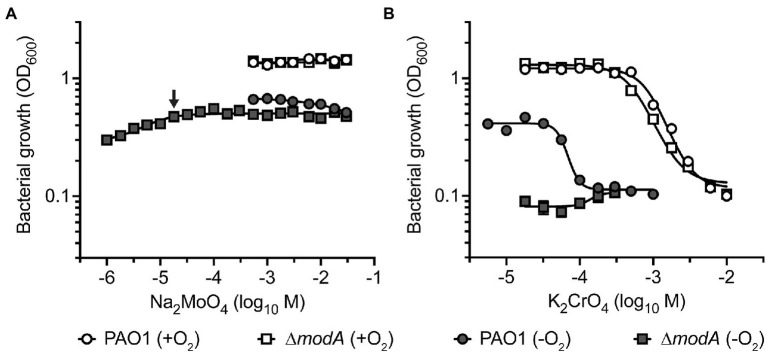
The impact of molybdate and chromate exposure on cell growth. Growth analysis of *P. aeruginosa* PAO1 wild-type and ∆*modA* strains after 24 h of aerobic (+O_2_) and anaerobic (−O_2_) growth in CDM media supplemented with increasing concentrations of **(A)** Na_2_MoO_4_ and **(B)** K_2_CrO_4_. One hundred millimolar KNO_3_ was added to CDM for anaerobic growth. Data corresponds to mean log_10_OD_600_ values ± SEM (*n* = 3). The concentration of Na_2_MoO_4_ required to restore optimal growth of the ∆*modA* strain in anaerobic conditions is indicated by an arrow. Where error bars are not visible, they are overlapped by symbols.

Building on the observed binding of CrO by ModA, we investigated whether exogenous CrO could perturb *P. aeruginosa* MoO uptake despite the substantial differences in relative oxyanion affinity determined *in vitro*. This was addressed by analyzing bacterial growth in cation defined media (CDM) supplemented with increasing concentrations of Na_2_MoO_4_ or K_2_CrO_4_. Supplementation of CDM with up to 30 mM Na_2_MoO_4_ had no significant effect on aerobic growth of the wild-type or ∆*modA* strains (Figure 5A). Anaerobic respiration *via* NarGHI-dependent nitrate reduction was assessed by growth in CDM with 100 mM KNO_3_. As observed in aerobic conditions, supplementation with up to 30 mM Na_2_MoO_4_ had no effect on anaerobic growth of the two strains (Figure 5A). However, growth of the ∆*modA* strain was dependent upon MoO supplementation in anaerobic conditions. Accordingly, the minimum Na_2_MoO_4_ concentration required for ∆*modA* anaerobic growth was determined in order to perform further investigations. We observed that the ∆*modA* strain required supplementation of 18 μM Na_2_MoO_4_ to restore optimal strain growth, which was defined as optical density values for bacterial growth comparable to growth in CDM supplemented with 30 mM Na_2_MoO_4_ (Figure 5A).

This framework was then used to investigate the impact of CrO on *P. aeruginosa* growth. Importantly, *P. aeruginosa* PAO1 does not encode the plasmid-borne CrO efflux system ChrA that has been reported in some clinical isolates (Cervantes and Ohtake, 1988; Cervantes et al., 1990). Supplementation of CDM with K_2_CrO_4_ (5.6 μM–10 mM) perturbed bacterial growth in a dose-dependent manner. In aerobic conditions, growth of the wild-type and ∆*modA* strains was abrogated by supplementation with 6 mM K_2_CrO_4_ (Figure 5B). Taken together, these data indicate that CrO exerts toxicity in aerobic conditions, and this occurs independent of ModA and/or MoO acquisition. Under anaerobic conditions, the potency of CrO toxicity was enhanced in the wild-type strain, showing inhibition of growth in 178 μM K_2_CrO_4_ (Figure 5B), which represents a 33.7-fold reduction in the minimum inhibitory concentration of the oxyanion. The ∆*modA* strain showed no growth in anaerobic conditions, irrespective of CrO supplementation, due to the low molybdate concentration of CDM (5 nM). These data suggest that despite sharing some chemical similarities, Cr cannot substitute for Mo and support nitrate reductase activity.

We then sought to ascertain whether CrO intoxication under anaerobic conditions could be overcome by MoO supplementation. Previous studies have shown that the impact of WO, a competitive inhibitor of MoO uptake, could be rescued by supplementation with equimolar concentrations of MoO (Pederick et al., 2014). Accordingly, given the 900-fold greater affinity of MoO for ModA over CrO, a growth-inhibitory concentration, such as 300 μM K_2_CrO_4_, should be rescued by a 100-fold excess of MoO. However, supplementation with up to 56 mM Na_2_MoO_4_ did not rescue growth (Supplementary Figure S5) indicating that the CrO-mediated abrogation of *P. aeruginosa* growth occurs, at least in part, independent of ModA and MoO acquisition. Accordingly, the cellular accumulation of metal ions was analyzed to determine the impact of exogenous K_2_CrO_4_ on cellular Mo levels.

### Exogenous Chromate Stress Dysregulates Molybdenum Homeostasis

**Figure 6:**
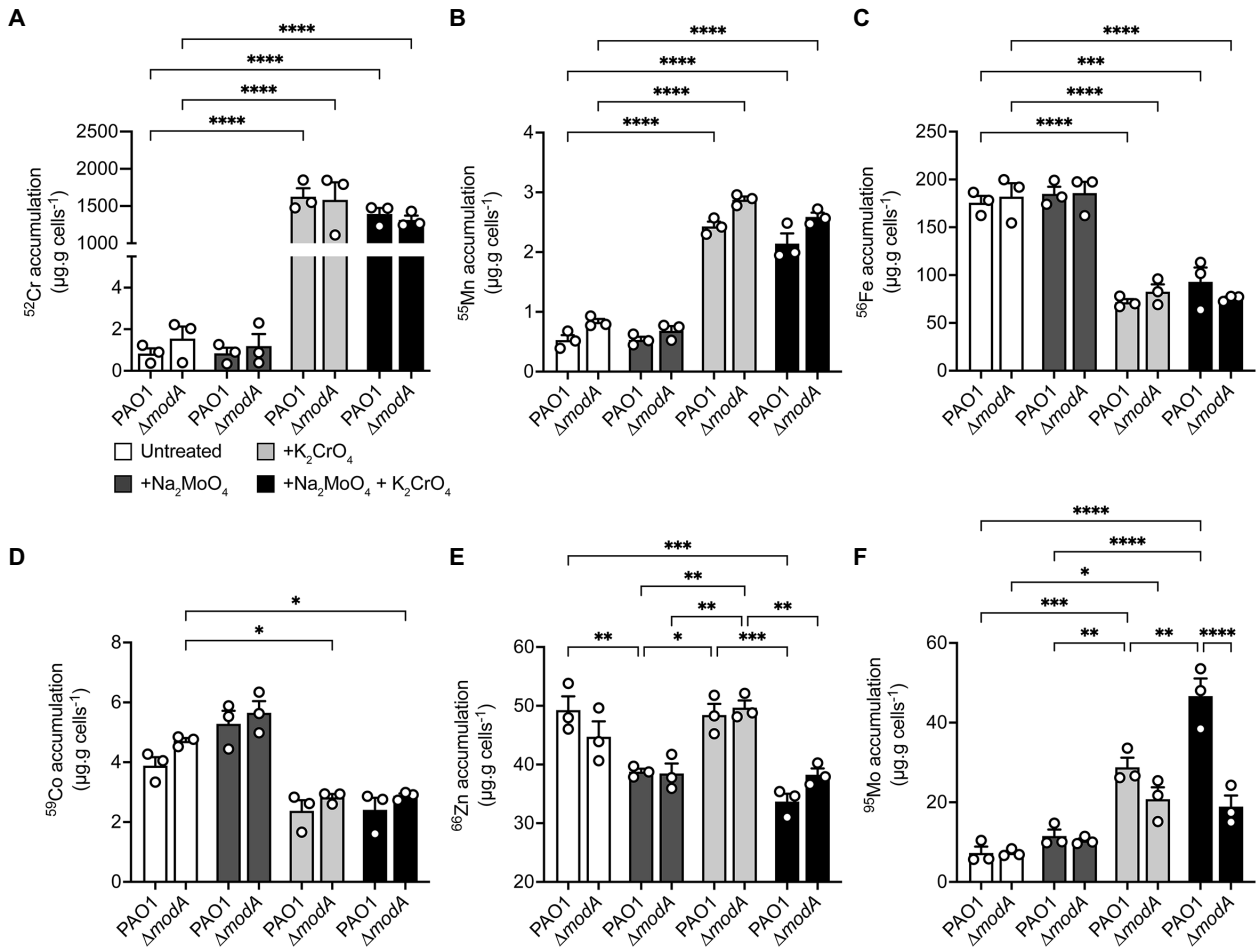
Modulations in cellular metal ion content under aerobic conditions in response to chromate and molybdate exposure. Whole cell accumulation of **(A)** chromium, **(B)** manganese, **(C)** iron, **(D)**, cobalt, **(E)** zinc, and **(F)** molybdenum in *P. aeruginosa* PAO1 wild-type and ∆*modA* strains grown aerobically in untreated M-CDM media or media supplemented with 320 μM Na_2_MoO_4_ and/or K_2_CrO_4_. Data represents the mean (±SEM) of three independent experiments. Statistical significance of the differences was determined by two-way ANOVA with Sidak post-test, * = *p* < 0.05, ** = *p* < 0.01, *** = *p* < 0.001, and **** = *p* < 0.0001.

**Figure 7:**
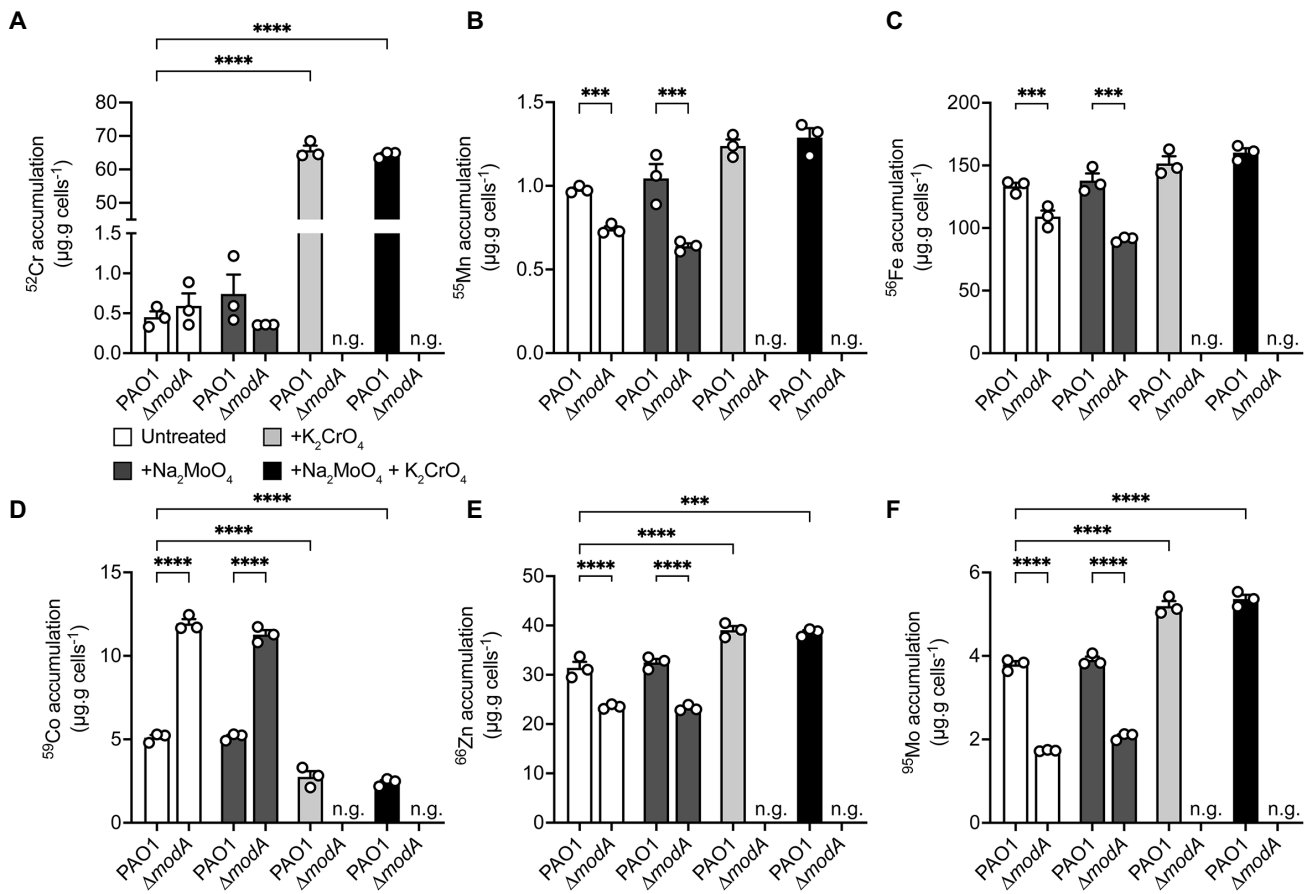
Modulations in cellular metal ion content under anaerobic conditions in response to chromate and molybdate exposure. Whole cell accumulation of **(A)** chromium, **(B)** manganese, **(C)** iron, **(D)**, cobalt, **(E)** zinc, and **(F)** molybdenum in the *P. aeruginosa* PAO1 wild-type and ∆*modA* strains grown anaerobically in untreated M-CDM media or media supplemented with 32 μM Na_2_MoO_4_ and/or K_2_CrO_4_. Media conditions where no bacterial growth was observed, denoted by n.g., are indicated. Data represents the mean (±SEM) of three independent experiments. Statistical significance of the differences was determined by two-way ANOVA with Sidak post-test, *** = *p* < 0.001 and **** = *p* < 0.0001.

Whole-cell metal content of the *P. aeruginosa* wild-type and ∆*modA* strains was determined using cultures grown in the presence or absence of equimolar concentrations of Na_2_MoO_4_ and/or K_2_CrO_4_ under aerobic (Figure 6) and anaerobic (Figure 7) conditions. Due to the influence of oxygen tension on the potency of CrO toxicity, sub-lethal concentrations of the metal, defined as 320 μM for aerobic or 32 μM for anaerobic conditions, were used. To ensure MoO sufficiency for the wild-type and Δ*modA* strains and thereby reveal CrO-induced depletion, if any, MoO-supplemented CDM (M-CDM) was prepared with the baseline Na_2_MoO_4_ concentrations of 100 μM (aerobic) and 10 μM (anaerobic).

In aerobic conditions, whole cell metal content analyses revealed that both the wild-type and Δ*modA P. aeruginosa* strains showed substantial ^52^Cr accumulation in CrO-supplemented M-CDM by comparison to untreated or MoO supplemented media (Figure 6A). However, no significant difference in cellular ^52^Cr levels between the wild-type and the Δ*modA* strain were observed. This indicated that ModA was not the major pathway of CrO uptake in M-CDM. Consistent with this inference, supplementation of M-CDM with equimolar concentrations of CrO and MoO did not reduce cellular ^52^Cr accumulation (Figure 6A). Supplementation of M-CDM with CrO or MoO+CrO was also associated with a significant increase in ^55^Mn accumulation (Figure 6B) and depletion of ^56^Fe (Figure 6C) in both strains. Although ^59^Co also showed reduced accumulation in both strains grown in CrO or MoO+CrO supplemented M-CDM, the reduction in accumulation was only statistically significant in the Δ*modA* strain (Figure 6D). Unexpectedly, M-CDM supplemented with MoO or MoO+CrO was associated with reduced cellular ^66^Zn accumulation in the wild-type and Δ*modA* strain (Figure 6E). Analysis of ^95^Mo accumulation in the wild-type and Δ*modA* strains showed that after growth in M-CDM, with or without additional MoO supplementation, both strains accumulated similar levels of the metal under aerobic conditions (Figure 6F). Supplementation of M-CDM with CrO resulted in a 3.9-fold increase in ^95^Mo accumulation in the wild-type strain, relative to the untreated condition, while treatment with equimolar MoO and CrO resulted in a 6.4-fold increase in ^95^Mo accumulation, relative to the untreated condition (Figure 6F). Interestingly, the increase in cellular ^95^Mo with equimolar MoO and CrO was not observed in the Δ*modA* strain. Thus, these data show that despite the capacity for ModA to interact with CrO, exposure to the metal and accumulation of ^52^Cr is associated with increased molybdate uptake. Further, CrO also dysregulates the homeostasis of ^55^Mn and ^56^Fe in both strains, and ^59^Co homeostasis in the Δ*modA* strain under aerobic conditions.

Under anaerobic conditions, M-CDM permitted growth of the wild-type and ∆*modA* strains. However, growth of the ∆*modA* strain was abolished upon addition of 32 μM CrO and could not be recovered by additional MoO supplementation of the M-CDM (Figure 7). In the wild-type strain, supplementation of M-CDM with CrO or MoO+CrO resulted in a significant increase in cellular ^52^Cr accumulation, relative to untreated and MoO supplemented M-CDM (Figure 7A). Interestingly, the dysregulatory effects of CrO exposure on ^55^Mn and ^56^Fe appeared to be abrogated under anaerobic conditions (Figures 7B,C). However, ^59^Co accumulation in the wild-type strain was significantly reduced, while ^66^Zn and ^95^Mo were significantly increased in response to CrO or MoO+CrO treatment (Figures 7D–F). In contrast, the ∆*modA* strain demonstrated significantly reduced cellular accumulation of ^55^Mn, ^56^Fe, ^66^Zn, and ^95^Mo, and increased ^59^Co accumulation in untreated and +MoO supplemented M-CDM relative to the wild-type strain (Figures 7B–F). Due to the CrO-mediated abrogation of *P. aeruginosa* ∆*modA* growth under anaerobic conditions, cellular accumulation of metals could not be determined in CrO or MoO+CrO supplemented M-CDM (Figure 7). Taken together, these data suggest that, even in the absence of CrO stress, the loss of *modA* mediates pleiotropic effects on *P. aeruginosa* metal homeostasis under anaerobic conditions. This finding is also consistent with the higher affinity of ModA for MoO over CrO and is distinct from the impact mediated by the group 6 oxyanion WO, which induced MoO depletion (Pederick et al., 2014). Notably, the potent impact of WO on MoO uptake is consistent with the similar affinities of ModA for the two oxyanions reported here (Table 2). Unexpectedly, the data here show that, under aerobic conditions, exogenous CrO induces an increase in cellular Mo that is largely attributable to the ModABC pathway. The increased accumulation of ^95^Mo also observed in the mutant strain, albeit to a lesser extent, in response to CrO exposure indicates that MoO is also acquired by secondary pathways. The ModA independent uptake of MoO may be attributable to transport by sulfur import pathways, which have also been shown to contribute to CrO accumulation (Holland and Avery, 2011), and further studies are warranted to identify the molecular basis thereof.

**Figure 8:**
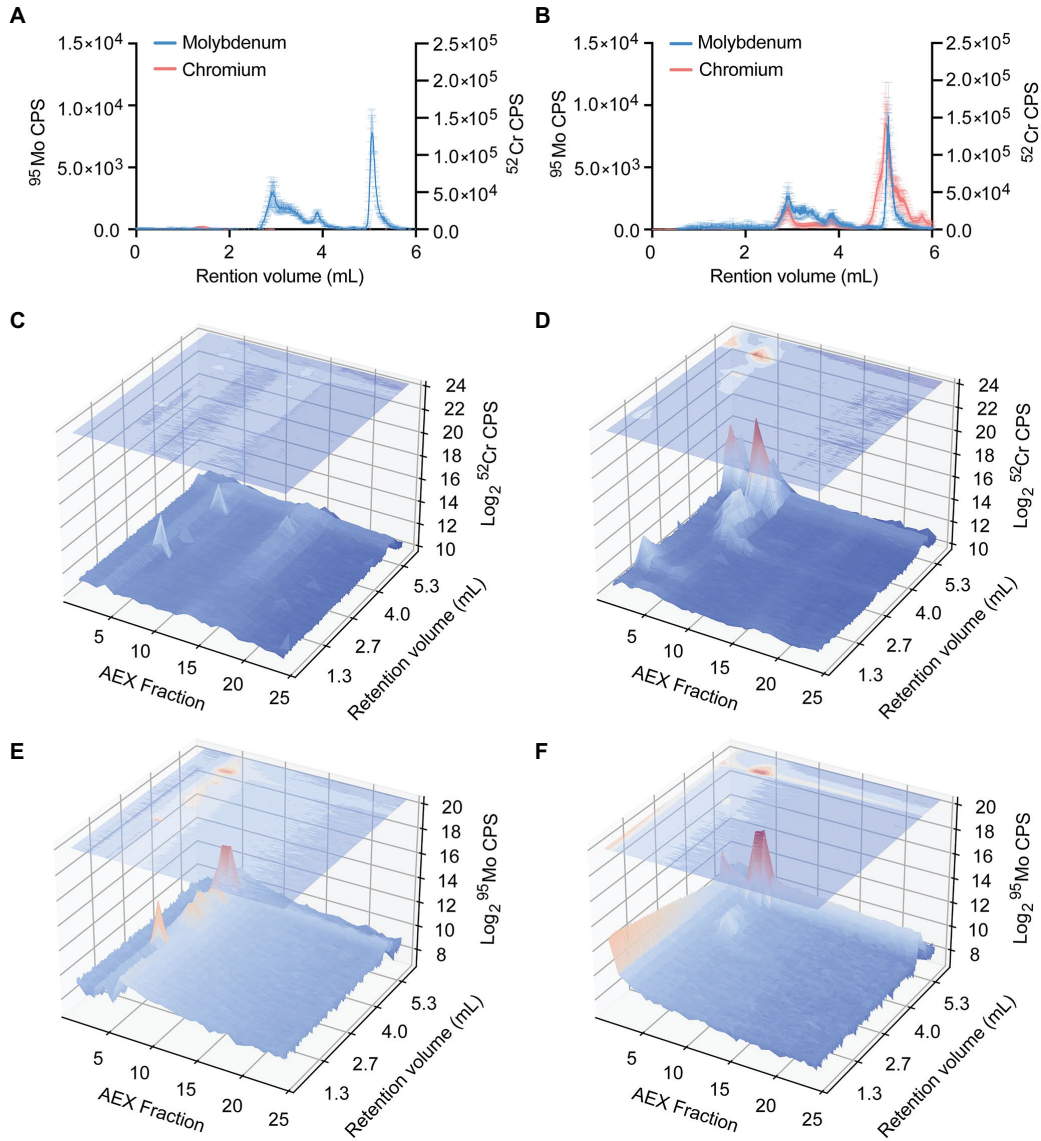
Distribution of chromium and molybdenum within *P. aeruginosa* cell lysates. Size exclusion chromatography (SEC)-ICP-MS chromatograms (counts-per-second, CPS) of chromium and molybdenum *P. aeruginosa* PAO1 lysates from cultures grown in **(A)** M-CDM and **(B)** M-CDM supplemented with 32 μM K_2_CrO_4_. Data represents the mean (±SEM) of three independent experiments. Representative *P. aeruginosa* lysates fractionated by anion exchange (AEX; *x*-axis) and further separated by SEC with online ICP-MS hyphenation (SEC; *y*-axis) overlayed over chromatograms (log_2_-CPS; *z*-axis) of chromium **(C,D)** and molybdenum **(E,F)**. Chromium chromatograms of *P. aeruginosa* lysates from cultures grown in **(C)** M-CDM, and **(D)** M-CDM supplemented with 32 μM K_2_CrO_4_. Molybdenum chromatograms of *P. aeruginosa* lysates from cultures grown in **(E)** M-CDM and **(F)** M-CDM supplemented with 32 μM K_2_CrO_4_.

The potent inhibition of anaerobic growth by CrO, despite the increased ^95^Mo accumulation, indicates that dissimilatory nitrate reduction is a target of CrO toxicity, either directly or indirectly. Accordingly, the impact of CrO on the distribution of ^95^Mo within the *P. aeruginosa* proteome was investigated by resolving bacterial lysates on the basis of size, using online hyphenated SEC-ICP-MS, and analyzing ^52^Cr and ^95^Mo content (Figure 8). SEC-ICP-MS analysis showed that exposure of *P. aeruginosa* to CrO during growth in M-CDM under anaerobic conditions led to the accumulation of ^52^Cr associated with macromolecular protein species with masses that overlapped with peaks containing ^95^Mo (Figures 8A,B). To further resolve the distribution of elements within the proteome, the bacterial lysate was fractionated by anion exchange (AEX) with the individual fractions then separated *via* SEC-ICP-MS to generate a two-dimensional elemental distribution map of the metalloproteome. This revealed the emergence of several ^52^Cr containing species within the cytoplasmic proteome, one of which appeared to overlap a ^95^Mo-containing species (Figures 8C–F). Further, the distribution of ^95^Mo-containing species decreased upon exposure to CrO (Figures 8E,F), despite an increase in the overall total cellular abundance, with the majority of ^95^Mo enriched within a single peak. These data show that CrO exposure disrupts cellular molybdenum distribution and metalloprotein binding. While specific ^95^Mo-bound species were not identified in these analyses, a global reduction in cellular molybdenum-associated proteins may manifest through disruption of molybdenum cofactor formation, and/or interference with maturation of molybdoenzymes, such as NarGHI, thereby impairing growth. Elucidation of the molecular targets of ^52^Cr intoxication and the impact on molybdoproteins warrants further investigation.

## Discussion

Molybdate acquisition is required for dissimilatory nitrate reduction and the production of energy under anaerobic conditions (Pederick et al., 2014). Recent studies have also highlighted the importance of MoO acquisition in *P. aeruginosa* virulence in a rat model of cystic fibrosis infection (Perinet et al., 2016), a murine model of acute pneumonia (Wang et al., 2021), its contribution to bacterial competition through type VI-dependent secretion of ModA (Wang et al., 2021), and in enhancing resistance to predation by *Dictyostelium discoideum* amoebae under aerobic conditions (Perinet et al., 2016). These observations indicate that MoO acquisition contributes to bacterial survival in both anaerobic and aerobic conditions, although the physiological requirement for the oxyanion under aerobic conditions remains to be defined. An analysis of two independent transcriptomic studies of *P. aeruginosa* infections in humans may provide some insight into this latter role (Cornforth et al., 2018; Elmassry et al., 2019). Those works revealed that only a single set of genes associated with a Mo-dependent protein complex was up-regulated during infection. In *P. aeruginosa* PAO1, the orthologous genes encode a putative Mo-dependent methionine sulfoxide reductase, MsrPQ, that has been shown to be crucial for protecting methionine containing periplasmic proteins against reactive oxygen and chlorine species, such as neutrophil generated hypochlorous acid, in *E. coli* and other bacterial species (Gennaris et al., 2015; Zhong et al., 2020). Thus, the diversity of ways in which Mo and the ModABC uptake pathway contribute to survival and virulence of *P. aeruginosa* has continued to expand and warrants further exploration.

The *modABC* operon is considered a core component of the *P. aeruginosa* PAO1 genome (Poulsen et al., 2019) and this work has shown that it is conserved in the majority of environmental and pathogenic *Pseudomonas* isolates analyzed. Although the nucleotide sequence of *modA* showed divergence across *Pseudomonas* spp., the amino acid sequence was moderately conserved. This is suggestive of negative selection pressures, which may be attributable to the intimate coupling of protein conformational changes with ligand-binding by SBPs (de Boer et al., 2019), such as *P. aeruginosa* ModA. The high-resolution metal-free and metal-bound crystal structures of *P. aeruginosa* PAO1 ModA provide detailed insights into the conformational landscape sampled during binding of the group 6 metal oxyanions CrO, MoO and WO. Similar to other cluster D-IIIa SBPs, *P. aeruginosa* ModA possesses a bilobal conformation with a single metal-binding site positioned within a deep cleft between the two lobes. The two lobes of *P. aeruginosa* ModA are hinged by two short, flexible cross-over segments, which enables increased movement between open and close conformations (Berntsson et al., 2010; Scheepers et al., 2016; Fukamizo et al., 2019). When the metal oxyanion is bound, this results in closure of the two lobes, corresponding to 22° relative rotation and occluding the bound oxyanion from bulk solvent by gating the metal-binding site with a hydrogen bond. The conformational transitions observed for *P. aeruginosa* ModA are consistent with the “Venus’ fly-trap” mechanism inferred for related MoO-recruiting SBPs (Hu et al., 1997; Balan et al., 2008). The mechanistic insights presented in this study highlight the benefit of directly comparing a single ModA protein in the metal-free and metal-bound state, which has not previously been reported to our knowledge. However, the absence of direct comparators precludes stronger conclusions regarding the extent of relative domain movement/rotation that arises as a consequence of ligand-binding by MoO-recruiting SBPs. Further, the contribution, if any, of the crystallization condition to the observed conformations of *P. aeruginosa* ModA should be also considered. Comparison of the metal-free and metal-bound *P. aeruginosa* ModA structures to orthologous ModA proteins in comparable states indicates that crystallization conditions (i.e., pH, salt, and other additives) do not appear to substantially influence the conformation of the protein (Hu et al., 1997; Lawson et al., 1997, 1998; Balan et al., 2008). Nevertheless, further structural analyses of ModA orthologues in both the metal-free and metal-bound state will aid in expanding our understanding of the extent and contribution of domain movements to SBP function.

Although *P. aeruginosa* ModA represents a canonical cluster D-IIIa fold, structural and biochemical characterization reveal subtle differences to previously studied SBPs. The three tetrahedral oxyanions are bound *via* six hydrogen bonds contributed by four binding residues: Asn33, Thr60, Tyr141 and Ile168. These oxyanion coordinating residues are conserved in almost all *Pseudomonas* spp. ModA proteins, supporting their crucial role in metal recruitment. The metal-binding site is flanked by β-strands, with the metal-coordinating residues contributed from surrounding α helices and βα loops. Notably, the β-strands also showed a high level of conservation most likely due to their role in maintaining the integrity of the binding site and protein function. The coordination chemistry observed in *P. aeruginosa* ModA has not previously been observed in orthologous ModA structures. To date, orthologous ModA proteins have been shown to bind oxyanions *via* seven hydrogen bonds coordinated by five binding residues (Hu et al., 1997; Lawson et al., 1997; Balan et al., 2008). Although *P. aeruginosa* ModA lacks this additional coordinating residue and hydrogen bond, the SBP has a nanomolar binding affinity for MoO and WO that is greater, up to ~30-fold, than other ModA orthologs studied to date (Rech et al., 1996; Imperial et al., 1998; Balan et al., 2006; Smart et al., 2009; Pederick et al., 2014). The higher affinity of *P. aeruginosa* ModA for MoO and WO may be attributable, at least in part, to the hydrogen bond that is formed between the metal-binding residues Asn33 and Tyr141 during the metal-binding process, although future studies would be required to ascertain the veracity of this inference.

The metal-binding site of *P. aeruginosa* ModA was shown to be capable of binding CrO, MoO, and WO, but showed no apparent interaction with smaller oxyanions, such as sulfate and phosphate. This can most likely be attributed to the relatively similar sizes of the group 6 oxyanions and preference for similar hydrogen bonding distances (Hu et al., 1997; Lawson et al., 1997; Balan et al., 2008). Nevertheless, ModA demonstrated a substantial preference, in terms of binding affinity and thermostability of the metal-bound complex, for MoO and WO relative to CrO. This may arise from access to the d-orbital shell of electrons in Mo and W, facilitating stronger hydrogen bonding interactions by comparison to CrO. Irrespective of the precise mechanism, *P. aeruginosa* ModA demonstrates a preference for acquiring MoO over CrO at physiologically relevant concentrations. However, other cluster D-IIIa SBPs, which coordinate oxyanion ligands in similar ways, may be more susceptible to inappropriately binding of CrO than *P. aeruginosa* ModA. For example, in *E. coli* ModA the relative affinity values for MoO and CrO differ by only ~2–4-fold (Rech et al., 1996; Imperial et al., 1998; Karpus et al., 2017; Ge et al., 2020) indicating that the SBP is likely to be more permissive to oxyanion competition than *P. aeruginosa* ModA. The combination of features in *P. aeruginosa* ModA, specifically nanomolar affinity and selectivity for MoO, may have evolved due to its unique roles in both periplasmic and extracellular recruitment of the oxyanion (Wang et al., 2021). This latter functionality of ModA is due to its type VI-dependent secretion from the periplasm into the extracellular milieu. Extracellular ModA has been reported to capture MoO in a small molecule, metallophore-independent manner and enable *P. aeruginosa* uptake of the oxyanion *via* IcmP (Wang et al., 2021). The dual roles of *P. aeruginosa* ModA in periplasmic and extracellular MoO recruitment aids in bacterial competition, but has not been reported for orthologous ModA proteins to date (Wang et al., 2021). By contrast, the inability of *P. aeruginosa* ModA to discriminate between MoO and WO is a common limitation in orthologous SBPs (Rech et al., 1996; Ge et al., 2020) and is consistent with the impact on Mo-dependent cellular functions (Pederick et al., 2014).

Deletion of *modA* abrogated MoO accumulation in Mo-limited growth medium. Despite this, the *P. aeruginosa* Δ*modA* strain acquired sufficient MoO for survival under anaerobic conditions in MoO-supplemented medium. Thus, this indicates that in addition to ModABC, there are secondary or non-specific oxyanion transporters, such as sulfate import pathways, that can facilitate MoO uptake. Although *P. aeruginosa* ModA has the potential to interact with CrO, phenotypic and molecular microbiological assays revealed it is not the primary route of uptake. This observation is consistent with the binding affinity and thermostability analyses of the SBP. Nevertheless, it is important to note that although deletion of *modA* did not affect cellular accumulation of Cr, we lack direct evidence for whether the ModBC transporter is permissive for CrO translocation. The most likely explanation for the observed Cr accumulation is that the CrO oxyanion is predominantly imported *via* pathways other than ModABC. The ability of CrO to be imported into bacteria, such as *P. fluorescens* and *Salmonella typhimurium*, *via* sulfate uptake pathways (Cervantes and Campos-Garcia, 2007), has been linked to the structural and chemical similarity of CrO to sulfate (Pardee et al., 1966; Ohtake et al., 1987).

Toxicity of CrO has been attributed to its high solubility and propensity to spontaneously reduce from the highly toxic Cr^6+^ cation to Cr^5+^, Cr^4+^, and Cr^3+^, in which free radicals are produced. Further redox cycling between Cr^5+^ and Cr^6+^ leads to the generation of ROS, which mediates damage to lipids, proteins, and DNA (Cervantes and Campos-Garcia, 2007; Cheung and Gu, 2007; Thatoi et al., 2014). However, in *P. aeruginosa*, CrO has a modest effect under aerobic conditions, whilst it is substantially more toxic under anaerobic conditions. It therefore follows that the molecular basis of toxicity is unlikely to be the generation of reactive oxygen species. Exposure to CrO disrupted the homeostasis of multiple distinct metal ions in both aerobic and anaerobic conditions, indicating that CrO toxicity is mechanistically complex. However, the potent impact of CrO on *P. aeruginosa* in anaerobic conditions shows that Mo-dependent processes are a key target. Exposure to CrO dysregulated Mo homeostasis, which manifested as an increase in cellular accumulation of the metal in a predominantly ModABC-dependent manner. This could be attributable to inappropriate interaction of Cr with ModE, the metalloregulator of the *modABC* operon. The formation of an unproductive Cr-ModE complex could result in increased *modABC* expression due to an inability to correctly sense cellular Mo levels. Alternatively, the master regulator Anr, which responds to oxygen limitation and can also influence *modA* expression, could have been indirectly impacted by CrO accumulation (Wang et al., 2021). Activity of Anr is regulated by the interaction of molecular oxygen with a labile [4Fe-4S]^2+^ cluster within the protein (Ibrahim et al., 2015). Prior studies have shown that CrO can compete with sulfur for uptake, and this could indirectly perturb Anr activity *via* disruption of iron–sulfur cluster formation. Irrespective of how Mo homeostasis was dysregulated, ^52^Cr accumulation abrogated the ability of the Δ*modA* strain to grow on nitrate, while survival of the wild-type strain was substantially compromised. This indicated that some aspect of dissimilatory nitrate reduction was perturbed. Examination of elemental distribution maps showed that CrO exposure reduced the complexity of the ^95^Mo proteome, with the metal largely restricted to a single peak. Thus, CrO exposure reduces molybdoprotein complexity within the proteome, which may be due to impairment of molybdenum cofactor assembly or disruption of molybdoenzyme maturation. The import pathway(s) for CrO in *P. aeruginosa*, the molecular targets of CrO toxicity, and the mechanistic basis for dysregulation of Mo homeostasis and molybdoprotein abundance warrants further study.

Collectively, these findings have provided insight into the limited specificity of ModA for group 6 oxyanions and have shown that ModA is implicated in the dysregulation of Mo homeostasis by excess CrO. These findings expand our understanding of how ModA recruits specific metal oxyanion ligands and how CrO intoxication perturbs growth of *P. aeruginosa* under aerobic and anaerobic conditions. This has potential environmental significance as CrO remains present in crustal and soil samples at up to 100-fold higher levels than Mo (Smith and Huyck, 1997). Thus, how bacteria discriminate between CrO and MoO may have relevance for future bioremediation applications.

## Data Availability Statement

The data present in the study are deposited in the Protein Data Bank, accession numbers: 7T4Z (metal-free ModA), 7T50 (chromate-bound ModA), 7T51 (molybdate-bound ModA), and 7T5A (tungstate-bound ModA).

## Author Contributions

EM contributed the mutant *P. aeruginosa* strains and the molecular microbiological analyses. DN contributed the structural analyses of ModA and the biophysical protein analyses. SH contributed the molecular dynamic analyses of ModA. KG contributed the inductively coupled plasma-mass spectrometry analyses and liquid chromatography-inductively coupled plasma-mass spectrometry. BL contributed to the structural analyses of ModA. ML contributed the data analysis workflow to generate multi-dimensional elemental plots. ZL contributed to the training and supervision of DN and BL for the structural analyses of ModA. AT contributed to the supervision of EM and the molecular microbiological analyses. ED contributed to the molecular dynamic analyses and supervised SH. BK contributed to the structural analyses and supervision of DN, BL, and ZL. CM contributed funding for the study, coordinated the research, supervised EM, KG, and AT, and wrote the manuscript. All authors contributed to the article and approved the submitted version.

## Funding

This work was supported by the National Health and Medical Research Council (NHMRC) grants 1071659 to BK, 1122582 to CM and 1180826 to BK and CM. BK is an ARC Laureate Fellow (FL180100109) and CM is an ARC Future Fellow (FT170100006).

## Conflict of Interest

The authors declare that the research was conducted in the absence of any commercial or financial relationships that could be construed as a potential conflict of interest.

## Publisher’s Note

All claims expressed in this article are solely those of the authors and do not necessarily represent those of their affiliated organizations, or those of the publisher, the editors and the reviewers. Any product that may be evaluated in this article, or claim that may be made by its manufacturer, is not guaranteed or endorsed by the publisher.
