# Supplementary material for: The Impact of Chromate on Pseudomonas aeruginosa Molybdenum Homeostasis
Source: Front Microbiol. 2022 May 24;13:903146. doi: 10.3389/fmicb.2022.903146 (PMC9171197; doi:10.3389/fmicb.2022.903146)
Supplement: Supplementary file 2 [file Data_Sheet_1.pdf]

SUPPLEMENTARY INFORMATION

Supplementary Table S1. Oligonucleotide primers used in this study.

Supplementary Table S2. Strains used in this study.

Supplementary Table S3. Plasmids used in this study.

Supplementary Table S4. X-ray crystallography data collection, processing, and refinement statistics.

Supplementary Table S5. Genomic screening of *Pseudomonas* spp.

Supplementary Figure S1. Structural comparison of crystal structures of metal-free and molybdate-bound *P. aeruginosa* ModA.

Supplementary Figure S2. Multiple sequence alignment of *P. aeruginosa* ModA with six bacterial ModA orthologues.

Supplementary Figure S3. Structural comparison of crystal structures of metal-free and metal-bound *P. aeruginosa* ModA with ModA orthologues.

Supplementary Figure S4. Structural comparison of metal-binding pockets of the crystal structures of metal-bound ModA orthologues.

Supplementary Figure S5. Growth analysis of CrO-intoxicated *P. aeruginosa* PAO1 with increasing concentrations of MoO.

Supplementary References

Supplementary Table S1. Oligonucleotide primers used in this study

Primer	Sequence (5'–3')	Comment
ModA_Fwd_23	TACTTCCAATCCAATGCAGACGAGGTGCAGGTCG	Cloning *modA* into pMCSG7 vector
ModA_Rev	TTATCCACTTCCAATGTTAGAGTTCGTAGCCGTAGGACTTG	Cloning *modA* into pMCSG7 vector

Supplementary Table S2. Strains used in this study

Bacterial strains	Genotype	Source
Escherichia coli BL21-Gold (DE3) competent	*E. coli* B F^- *dcm*+ Hte *ompT hsdS*(r_B^- m_B^-) *gal* λ (DE3) *endA* Tet^r	Agilent Technologies
Pseudomonas aeruginosa PAO1	Laboratory strain	(Pederick et al., 2014)
Pseudomonas aeruginosa PAO1 Δ*modA*	Laboratory strain with null mutant of *modA*	(Pederick et al., 2014)

Supplementary Table S3. Plasmids used in this study

Plasmid	Description	Source
pMCSG7	Amp^R, ligation-independent cloning protein expression vector encoding a N-terminal hexahistidine fusion tag.	This study
pCAM-cLIC01	Kan^R, ligation-independent cloning protein expression vector encoding a C-terminal dodecahistidine fusion tag.	(Pederick et al., 2014)
pMCS7-*modA*	Amp^R, *modA* gene lacking the signal sequence (residues 1 to 22), cloned into the pMCSG7 vector	This study
pCAM-cLIC01-*modA*	Amp^R, *modA* gene lacking the signal sequence (residues 1 to 23), cloned into the pCAM-cLIC01 vector	(Pederick et al., 2014)

Supplementary Table S4. X-ray crystallography data collection, processing, and refinement statistics

**Data collection**				
**Protein**	**Metal-free ModA**	**Chromate-bound ModA**	**Molybdate-bound ModA**	**Tungstate-bound ModA**
**Diffraction source**	Australian Synchrotron, MX2 Beamline	Australian Synchrotron, MX2 Beamline	Australian Synchrotron, MX1 Beamline	Australian Synchrotron, MX2 Beamline
**Wavelength (Å)**	0.954	0.954	0.954	0.954
**Resolution range (Å)**	46.68 – 1.78 (1.82 – 1.78)	45.57 – 1.90 (1.94 – 1.90)	45.48 – 2.50 (2.60 – 2.50)	46.07 – 2.16 (2.23 – 2.16)
**Temperature (K)**	100	100	100	100
**Rotation range per image (°)**	0.10	0.10	0.10	0.10
**Total rotation range (°)**	180.00	180.00	90.00	420.00
**Space group**	$C\ 2\ 2\ 2_1$	$P\ 1\ 2_1\ 1$	$P\ 1\ 2_1\ 1$	$P\ 1\ 2_1\ 1$
**a, b, c (Å)**	106.49, 128.91, 67.69	45.63, 41.22, 111.37	45.53, 40.90, 111.09	46.11, 40.98, 111.04
**α, β, γ (°)**	90.00, 90.00, 90.00	90.00, 92.76, 90.00	90.00, 92.54, 90.00	90.00, 92.53, 90.00
**Mosaicity (°)**	0.07	0.11	0.41	0.38
**Completeness (%)**	99.9 (98.2)	99.1 (94.7)	94.7 (71.5)	99.8 (99.5)
**CC (1/2)**	0.999 (0.872)	0.996 (0.687)	0.993 (0.906)	0.994 (0.715)
**CC***	1.000 (0.972)	0.999 (0.917)	0.998 (0.969)	0.998 (0.909)
**R_{merge} (%)**	6.7 (51.8)	10.3 (75.1)	8.2 (24.9)	17.9 (88.6)
**R_{meas} (%)**	7.3 (56.2)	12.2 (88.8)	9.9 (30.7)	19.3 (94.0)
**R_{pim} (%)**	2.8 (21.4)	6.5 (46.9)	5.4 (17.7)	7.0 (35.9)
**$\langle I/\sigma(I) \rangle$**	16.7 (3.5)	8.6 (1.7)	10.6 (3.8)	9.5 (2.3)
**Multiplicity**	6.8 (6.7)	3.4 (3.4)	3.2 (2.4)	7.4 (6.5)
**Total no. of reflections**	304329 (16502)	111545 (6802)	43587 (2781)	166918 (12702)
**No. of unique reflections**	44640 (2473)	32791 (1972)	13730 (1155)	22601 (1945)
**Wilson B-factor ($Å^2$)**	21.64	22.13	22.70	24.01
**Refinement**				
**No. of reflections used**	44620 (4354)	32744 (3227)	13684 (1019)	22564 (2224)
**R_{work} (%)**	18.72 (26.60)	23.35 (29.34)	25.89 (31.14)	22.96 (28.00)
**R_{free} (%)**	21.09 (31.90)	26.65 (30.27)	28.89 (44.87)	26.37 (32.65)
**Ramachandran plot**				
**Favored (%)**	98.9	98.2	96.4	97.3
**Allowed (%)**	1.1	1.8	3.3	2.7
**Outliers (%)**	0	0	0.2	0

No. of non-hydrogen atoms				
**Protein**	3426	3393	3390	3424
**Ligands**	29	81	29	30
**Water**	278	218	52	136
**Average B-factors (Å^2)**				
**Protein**	24.97	26.52	27.78	29.85
**Ligands**	38.45	26.81	20.07	21.70
**Waters**	32.84	32.77	27.18	27.46
**R.m.s. deviations**				
**Bond lengths $_{r.m.s.}$ (Å)**	0.008	0.009	0.008	0.019
**Bond angles $_{r.m.s.}$ (°)**	0.97	0.97	1.23	1.31

1. The statistics shown are based on the calculations using *Aimless* (Evans and Murshudov, 2013) and *Molprobity* (Chen et al., 2010).

2. The statistics for the highest resolution shell are shown in parentheses.

3. $R_{merge} = \sum_{hkl} \sum_j |I_{hkl,j} - < I_{hkl} > | / (\sum_{hkl}\sum_j I_{hkl,j})$; $R_{meas} = \sum_{hkl} [N/(N-1)]\ 1/2 \sum_j |I_{hkl,j} - < I_{hkl} > | / (\sum_{hkl} \sum_j I_{hkl,j})$; $R_{pim} = \sum_{hkl} [1/(N-1)]\ 1/2 \sum_j |I_{hkl,j} - < I_{hkl} > | / (\sum_{hkl} \sum_j I_{hkl,j})$

4. $R_{work} = \sum_{hkl} |Fobs_{hkl} - Fcalc_{hkl}| / \sum|Fobs_{hkl}|$; R_{free} is equivalent to R_{work}, with 5% of data excluded from refinement process. $|Fobs_{hkl}|$ and $|Fcalc_{hkl}|$ represent the observed and calculated structure factor amplitudes.

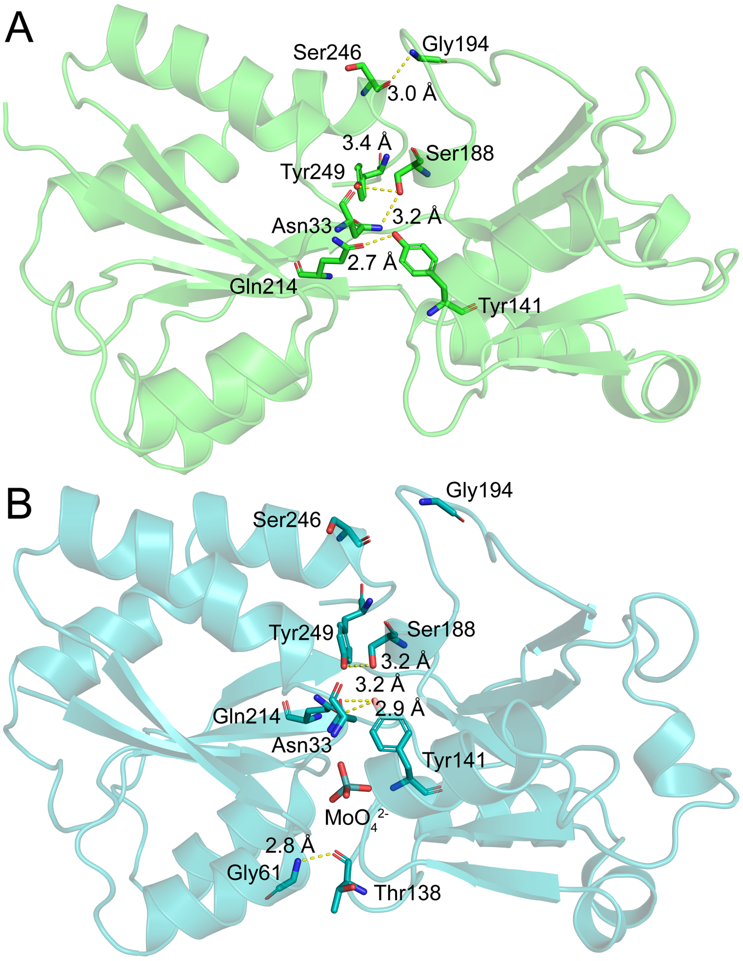

Supplementary Figure S1. Structural comparison of crystal structures of metal-free and molybdate-bound *P. aeruginosa* ModA.

The crystal structures of (**A**) metal-free (green) and (**B**) molybdate-bound (teal) *P. aeruginosa* PAO1 ModA are shown in cartoon representation. Residues involved in interaction and stabilization of the two lobes are labelled and shown in stick representation. The molybdate oxyanion is shown in stick representation and labelled. The coordination bonds are shown as yellow dotted lines and labelled with their respective bond lengths. The nitrogen and oxygen atoms are colored blue and red, respectively.

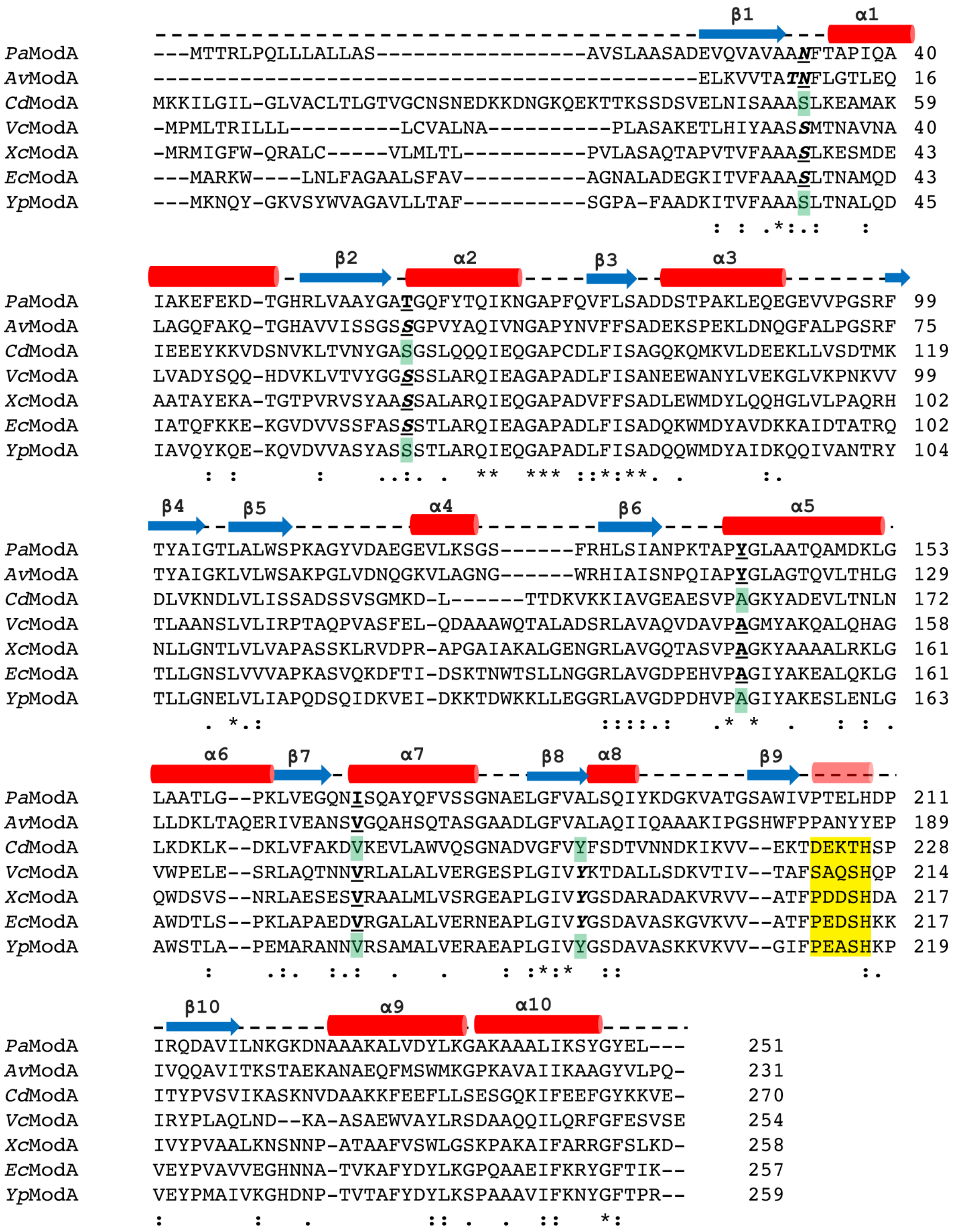

Supplementary Figure S2. Multiple sequence alignment of *P. aeruginosa* ModA with six bacterial ModA orthologues.

A multiple sequence alignment of *P. aeruginosa* PAO1 ModA (*Pa*ModA) with six bacterial ModA orthologues with available crystal structures was constructed using the Clustal Omega 2.1 webtool from the EMBL-EBI website (Madeira et al., 2019). The selected sequences include the *Azotobacter vinelandii* ModA (*Az*ModA, 46% sequence identity, UniProt ID: Q7SIH2); *Xanthamonas axonopodis* pv. *citri* ModA (*Xc*ModA, 29% sequence identity, UniProt ID: Q8PHA1), *Escherichia coli* ModA

(*Ec*ModA, 27% sequence identity, UniProt ID: P37329), *Vibrio cholerae* serotype 01 ModA (*Vc*ModA, 26% sequence identity, UniProt ID: Q9KLL7); *Yersinia pestis* ModA (*Yp*ModA, 26% sequence identity, UniProt ID: Q8D002), and *Clostridioides difficile* ModA (*Cd*ModA, 25% sequence identity, UniProt ID: Q18A64). The positions of the α helices and β strands in *Pa*ModA are represented by cylinders and arrows, respectively. The residues in bold are involved in ligand binding, with those italicized involved in side-chain interaction and those underlined involved in main-chain interaction. Because the published structures of *Yp*ModA and *Cd*ModA correspond to ligand-free forms, the residues proposed to be involved in ligand-binding based on conserved residues are highlighted in green. The residues in grey are the interdomain linkers. The residues highlighted in yellow correspond to an additional α helix present between the strands β9 and β10 for all structures except *Pa*ModA and *Az*ModA. The '*' symbol represents fully conserved residues. The '.' and ':' symbols represent residues with "similar" and "more similar" properties, respectively.

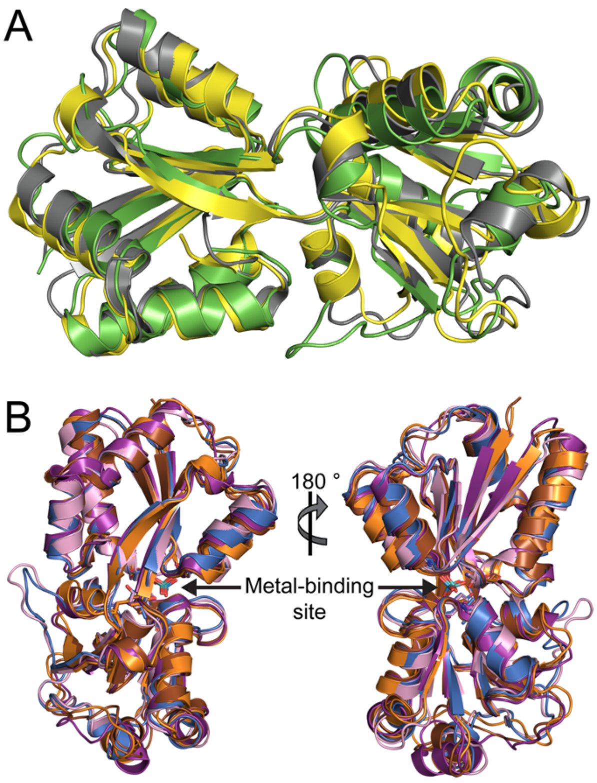

Supplementary Figure S3. Structural comparison of crystal structures of metal-free and metal-bound *P. aeruginosa* ModA with ModA orthologues.

(**A**) Structural superposition of metal-free crystal structures of *P. aeruginosa* PAO1 ModA (green), *Y. pestis* ModA (yellow) and *C. difficile* ModA (grey) in cartoon representation. (**B**) Structural superposition of metal-bound crystal structures of *P. aeruginosa* PAO1 ModA (teal), *A. vinelandii* ModA (pink), *X.* pv. *citri* ModA (brown), *E. coli* ModA (orange) and *V. cholerae* ModA (purple) in cartoon representation. The metal oxyanions and residues involved in metal-binding are shown in stick representation.

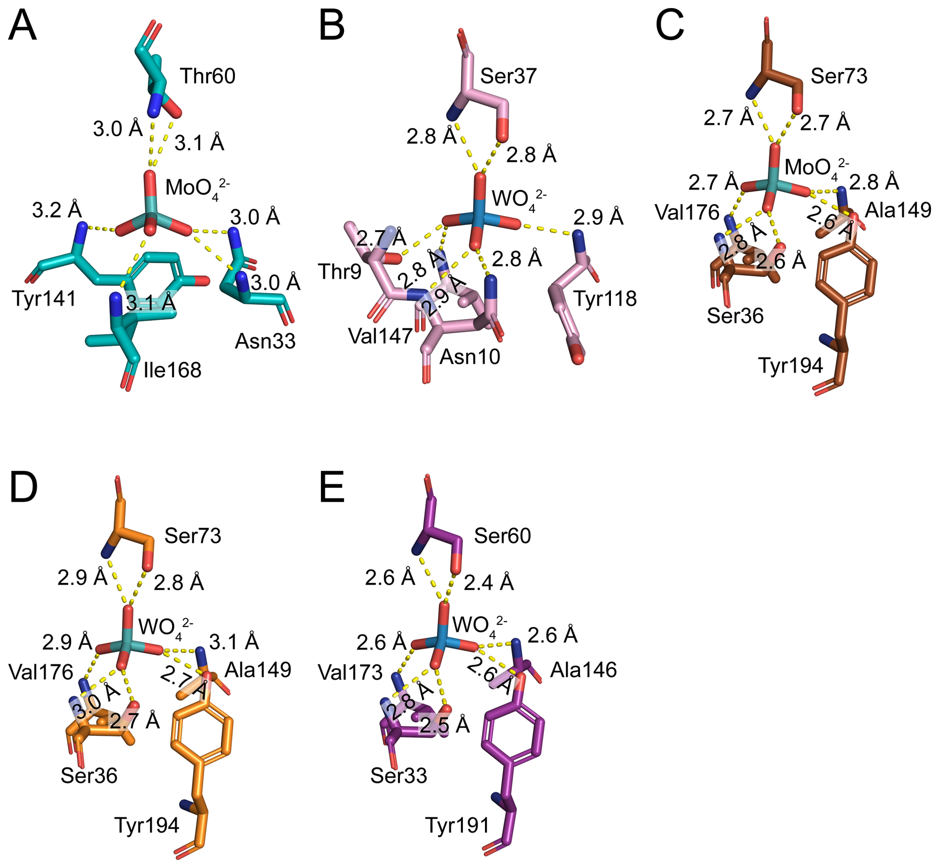

Supplementary Figure S4. Structural comparison of metal-binding pockets of crystal structures of metal-bound ModA orthologues.

The ligand-binding pockets of metal-bound (**A**) *P. aeruginosa* PAO1 ModA (teal), (**B**) *A. vinelandii* ModA (pink) (PDB ID: 1ATG), (**C**) *X.* pv. *citri* ModA (brown) (PDB ID: 2H5Y), (**D**) *E. coli* ModA (orange) (PDB ID: 1AMF) and (**E**) *V. cholerae* ModA (purple) (PDB ID: 4RXL) are shown in stick representation. The metal oxyanion ligands and residues involved in metal-binding are shown in stick representation and labelled. The coordination bonds are shown as yellow dotted lines and labelled with their respective bond lengths. The nitrogen and oxygen atoms are colored blue and red, respectively.

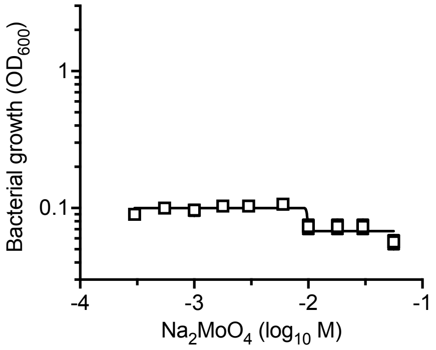

Supplementary Figure S5. Impact of molybdate supplementation on chromate intoxication.

Growth analysis of *P. aeruginosa* PAO1 after 24 h of anaerobic growth in CDM media supplemented with an inhibitory concentration of K_2CrO_4 (300 μM) and increasing concentrations of Na_2MoO_4 (0.31 - 56 mM). Data corresponds to mean log_{10} OD_{600} values (± SEM) with experiments conducted in biological triplicate. Where error bars are not visible, they are overlapped by the symbols.

Supplementary References

Chen, V.B., Arendall, W.B., 3rd, Headd, J.J., Keedy, D.A., Immormino, R.M., Kapral, G.J., Murray, L.W., Richardson, J.S., and Richardson, D.C. (2010). MolProbity: all-atom structure validation for macromolecular crystallography. *Acta Crystallogr D Biol Crystallogr* 66**,** 12-21.

Evans, P.R., and Murshudov, G.N. (2013). How good are my data and what is the resolution? *Acta Crystallogr D Biol Crystallogr* 69**,** 1204-1214.

Madeira, F., Park, Y.M., Lee, J., Buso, N., Gur, T., Madhusoodanan, N., Basutkar, P., Tivey, A.R.N., Potter, S.C., Finn, R.D., and Lopez, R. (2019). The EMBL-EBI search and sequence analysis tools APIs in 2019. *Nucleic Acids Res* 47**,** W636-W641.

Pederick, V.G., Eijkelkamp, B.A., Ween, M.P., Begg, S.L., Paton, J.C., and McDevitt, C.A. (2014). Acquisition and role of molybdate in *Pseudomonas aeruginosa*. *Appl Environ Microbiol* 80**,** 6843-6852.
